# Novel Therapeutic Nutrients Molecules That Protect against Zika Virus Infection with a Special Note on Palmitoleate

**DOI:** 10.3390/nu15010124

**Published:** 2022-12-27

**Authors:** Philma Glora Muthuraj, Chandan Krishnamoorthy, Ann Anderson-Berry, Corrine Hanson, Sathish Kumar Natarajan

**Affiliations:** 1 Department of Nutrition and Health Sciences, University of Nebraska-Lincoln, Lincoln, NE 68583, USA; 2 Child Health Research Institute, University of Nebraska Medical Center, Omaha, NE 68198, USA; 3 Department of Pediatrics, University of Nebraska Medical Center, Omaha, NE 68198, USA; 4 Medical Nutrition Education, College of Allied Health Profession, University of Nebraska Medical Center, Omaha, NE 68198, USA

**Keywords:** apoptosis, placenta, ER stress, congenital Zika syndrome

## Abstract

Zika virus (ZIKV) is a Flavivirus from the Flaviviridae family and a positive-sense single strand RNA virus. ZIKV infection can cause a mild infection to the mother but can be vertically transmitted to the developing fetus, causing congenital anomalies. The prevalence of ZIKV infections was relatively insignificant with sporadic outbreaks in the Asian and African continents until 2006. However, recent epidemic in the Caribbean showed significant increased incidence of Congenital Zika Syndrome. ZIKV infection results in placental pathology which plays a crucial role in disease transmission from mother to fetus. Currently, there is no Food and Drug Administration (FDA) approved vaccine or therapeutic drug against ZIKV. This review article summarizes the recent advances on ZIKV transmission and diagnosis and reviews nutraceuticals which can protect against the ZIKV infection. Further, we have reviewed recent advances related to the novel therapeutic nutrient molecules that have been shown to possess activity against Zika virus infected cells. We also review the mechanism of ZIKV-induced endoplasmic reticulum and apoptosis and the protective role of palmitoleate (nutrient molecule) against ZIKV-induced ER stress and apoptosis in the placental trophoblasts.

## 1. Introduction

Zika virus (ZIKV) is a *Flavivirus* and was first isolated in a sentinel monkey kept for studying mosquito-borne diseases, and was also later isolated from *Aedes africanus* mosquitoes, confirming its vector-borne transmission, in the Ziika forest of Uganda [1]. Intracerebral inoculations of ZIKV in young mice showed extensive neurological lesions, while inoculation from the mice to non-human primates resulted in a self-limiting febrile condition in a few subjects. Later, neutralizing antibodies were found in both humans and monkeys on serological screenings [2]. The first human case was reported during the isolation process of the virus, wherein the clinical signs were described as pyrexia along with rashes by Simpson et al. in 1964 [3]. The occurrence of neurological abnormalities in infants born to pregnant mothers infected with ZIKV created concern regarding disease outbreak [4]. Currently, there is no Food and Drug Administration (FDA) approved vaccine or treatment for ZIKV infection. In the following section, we have reviewed recent advances related to novel nutrient molecules and compounds that show promising therapeutic applications for treating ZIKV infection.

## 2. ZIKV Epidemiology

The first widespread cluster of ZIKV outbreaks was reported from Yap Island in Micronesia [5]. Around 2013–2014, another outbreak with a considerable number of infections occurred in French Polynesia [6]. Reports of various modes of transmission other than mosquitoes and involvement of neurological disorders such as Guillain-Barre syndrome in a subset of the population were also observed during this outbreak [7,8]. The presence of vectors and travel-related introductions of ZIKV to a population without any prior exposure, along with other existing arboviral infections such as dengue and Chikungunya, may have favored the increased transmission of disease observed in the recent outbreaks [9,10,11,12]. In 2015 ZIKV had spread to Brazil, and later ZIKV spread to other parts of the American continent including Colombia, Honduras, Puerto Rico, the Dominican Republic, Jamaica, and Haiti [13]. In the mainland of the United States of America (USA), cases were also reported in the state of Florida in 2016 [14]

### 2.1. ZIKV Strains

Genetic changes in the ZIKV, involving complex interactions between the vector, human populations and non-human primate populations led to the evolution of the virus [15]. Two lineages of ZIKV are (1) Asian origin and (2) African origin. The African strain has two groups, the Ugandan versus the Nigerian group. The strain originally isolated from Rhesus macaque in the Ziika forest is MR-766, whereas IbH is the first strain isolated from the human blood in Nigeria [16]. The first isolated Asian ZIKV strain is from Malaysia with the prototype strain P6-740, and the cluster includes strains from Cambodia, French Polynesia and other Asian countries. In addition, some reports describe that the African strain is more cytotoxic to placental cells than Asian strains but both strains showed similar replicative efficiency [17]. 

ZIKV strains in the American continent that circulated from the 2015–2016 Brazil outbreak, evolved from the Asian lineage [18]. Travel-related to major sports events could have contributed to the spread of the virus from Pacific islands including French Polynesia to Brazil [19]. The presence of a new glycosylation motif in an asparagine residue at position 154 of envelope protein in the 2007 Yap strain- EC Yap and the French Polynesian H/PF/2013 strain could possibly explain the gain in their virulence when compared to MR766 which does not have this glycosylation motif [8]. 

### 2.2. Transmission of ZIKV

Usually, the disease is spread by the bite of the infected mosquito (*Aedes aegypti, Aedes albopictus*) [20]. The infection can also be vertically transmitted from infected mother to fetus. It can also be sexually transmitted, as ZIKV RNA is detected in semen samples of infected patients even after 6 months of infection [21,22], although only 3% of the total ZIKV cases account for sexually transmitted cases and a study suggests that semen suppresses the binding of ZIKV to cells [23]. Blood transfusions from infected individuals could also be a potential source of ZIKV infection early in the epidemic [24]. The virus replicates in the epithelial cells of the mosquitoes’ gut and later spreads to the mosquitoes’ salivary gland: then, the virus spreads to humans via a mosquito bite [25,26]. The receptors in the dermal fibroblasts, immature dendritic cells and keratinocytes facilitate viral entry and support viral replication [27]. Wild macaques are naturally susceptible to ZIKV infection [28]. The arbovirus infection follows a sylvatic cycle with non-human primates as the reservoir of the virus [29]. They serve as the connecting bridge for ZIKV circulation among mosquitoes and transmission to humans due to the extensive urbanization in the present-day scenario [30]. 

### 2.3. ZIKV Structure

ZIKV is icosahedral in symmetry, ~40 nm with a nucleocapsid ~25–30 nm and surface projections ~5–10 nm [31,32]. Its genome is 10.8 Kb with 5′ NCR (translation via a methylated nucleotide cap or a genome-linked protein) and 3′ NCR (translation, RNA packaging, cyclization, genome stabilization and recognition)[33,34,35]. The virion consists of an envelope (E protein) covering the majority of the surface with non-structural proteins NS1 for virion production, NS3, and NS5 are large, highly conserved proteins, NS2A, NS2B, NS4A, and NS4B are small, hydrophobic proteins and NS4B, NS5 are targets for evolution [35,36,37]. Functions of individual ZIKV proteins are enlisted in Table 1.

### 2.4. ZIKV Replication

**Figure 1:**
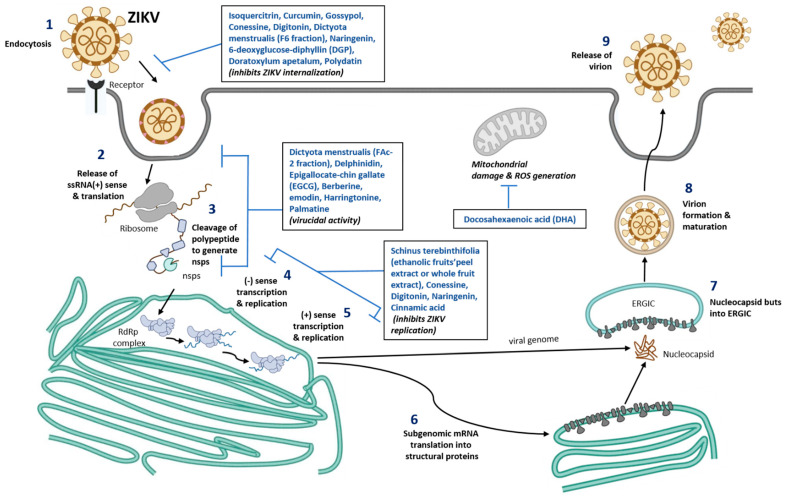
Schematic representation of nutraceuticals role in blocking ZIKV replication. Number 1–9 represent different stages of ZIKV infection, viral assembly, new viral formation, and release of mature virus from the infected cells. Nutrients compound identified that are known to inhibit various stages of viral infection are listed in the box inserts. ssRNA, single stranded ribonucleic acid; ERGIC, ER-Golgi intermediate compartment; RdRp, RNA dependent RNA polymerase; ROS, Reactive oxygen species; nsps, non-structural proteins of Zika virus.

Virus entry into the cell occurs by the initial recognition of host receptors by glycosylated regions on the envelope protein of the ZIKV [48]. Endocytosis of the infectious viral particle occurs by clathrin-coated vesicles. A low pH environment within the endosome facilitates conformational changes in the envelope protein of the virus, resulting in fusion to the endosome and thereby releasing the positive-strand RNA of the virus [47]. The positive strand becomes translated in the endoplasmic reticulum of the host cells into a polyprotein that is cleaved by the host cell proteases and the viral non-structural proteins such as NS3 and NS2B, which is a co-factor for protease. Non-structural proteins NS5 (RNA-dependent RNA polymerase) and NS3 (helicase) also replicate the positive-sense RNA strand to form a negative-sense RNA strand [49]. The negative-sense RNA strand serves as a template for further production of a new positive sense RNA strand. The newly produced positive sense RNA strand can either be translated or further used for viral genome replication [50]. After the assembly of structural proteins around the viral genome, they are translocated to the Golgi apparatus where they become mature virions by cleavage of the precursor membrane protein and exit the host cell [51] (Figure 1).

### 2.5. Clinical Findings and Congenital Zika Syndrome

In normal healthy children and adults, ZIKV infection usually presents with a mild febrile disease with rashes and joint pain [52]. Pregnant women typically develop symptoms such as rashes during ZIKV infection [53]. ZIKV infection in pregnant women results in both congenital brain defects and ocular defects in the fetus. Brain defects include microcephaly, cerebral atrophy, subcortical calcifications, agyria, hydrocephalus and ventriculomegaly [54]. Ocular defects include microphthalmia, optic nerve defects, cataract, and intraocular calcifications. Congenital contractures, reduced musculoskeletal movements, dysphagia, hypertonia, hypotonia, seizures and irritability are also reported in infants with in utero ZIKV infection [55]. Further, a case-control study showed that women with ZIKV infection during the early stages of pregnancy were more likely to have babies with congenital Zika syndrome (CZS) [56]. ZIKV infection is also associated with the development of Guillain-Barre syndrome in some adults, which is an autoimmune condition affecting the nervous system [57].

### 2.6. Diagnosis, Treatment, and Prevention of ZIKV Infection

In suspected ZIKV cases, a diagnosis is usually based on laboratory confirmation using IgM detecting serological test or RT-PCR based on E and NS2B genes [58,59,60]. In a particular place when there are ongoing outbreaks, it is recommended for pregnant women to be tested for ZIKV infection [61]. Serology tests can detect ZIKV as early as one week after suspected infection, but cross-reacting antibodies from other Flaviviruses can result in false-positive serological results [62]. Measuring viral RNA copy number using RT-PCR can also be used to detect the initial viremia in urine samples, cord blood and placental samples at delivery [63].

Currently, there is no approved vaccine for the effective prevention of the disease [64]. Only supportive treatment is available if infected [65]. Implementing effective mosquito control strategies in places with ZIKV infection is crucial to break the chain of ongoing disease spread [66]. Avoiding travel to areas with ongoing ZIKV outbreaks, especially if pregnant or planning to become pregnant are some of the ways to reduce the risk of infection [67]. There us ab option of using genetically modified *Aedes aegypti* mosquitoes to reduce the population of wild type mosquitoes to control mosquito-borne disease, but it is considered an emerging risk [68]

### 2.7. ZIKV Vaccines and Drug Development

ZIKV vaccine development is challenged by the target audience; it must be safe for pregnant women and to prevent neurological disorders in adults and fetuses [69,70]. Despite the challenges, several vaccine candidates have entered preclinical animal studies and phase I clinical trials. Some of the vaccine candidates which have entered phase I clinical trials that are noteworthy to mention include DNA vaccines by Inovio Pharmaceuticals and NIH, whole purified inactivated vaccine by WRAIR/Sanofi Pasteur Limited and Live, Dengue virus vectored vaccine by Butantan Institute [69]. Another major issue in vaccine development and translation of the vaccine technology into use is that ZIKV outbreaks had waned, making it too challenging to test the effectiveness of the vaccine without ongoing active disease transmission, along with the slow decline in funding which supports vaccine development [71]. Several drug repurposing studies have been conducted and found to be effective against ZIKV infection. However, there are no FDA-approved drugs available for ZIKV infection because most of the drugs do not have enough data to support safety in pregnant women. Examples of existing drugs with anti-ZIKV activity are suramin, nitazoxanide, chloroquine (anti-protozoal drugs), niclosamide, ivermectin (anthelmintics), mycophenolic acid (an immunosuppressant drug), PHA-690509 (cyclin-dependent kinase inhibitor) and sofosbuvir (an anti-viral drug effective against hepatitis C virus, [72]. Sofosbuvir has shown promising results in preventing ZIKV transmission from mother to fetus in pregnant mice and pregnant non-human primate models [73,74]. Interestingly, for some phytochemical compounds a computation approach shows that Polydatin, Liquiritin, Cichoriin, Dihydrogenistin and Rhapontin shows high docking score compared to the Sofosbuvir. Especially, Polydatin has more capacity for receptor binding when compared to Sofosbuvir (Table 2). Thus, phytochemicals can be used as a cost-effective ZIKV inhibitors; however, biocompatibility and effectiveness have to be proved in non-computational research experiments [75].

## 3. Nutraceuticals against ZIKV Infection

Nutraceuticals are naturally occurring compounds in food with health or medicinal value [76]. In an insilico analysis, around 2263 plant-derived compounds were screened and 43 of those compounds had anti-viral potential against ZIKV. Some of the well-known plant-derived compounds which could bind to ZIKV proteins are kanzonol V from licorice root (*Glycyrrhiza glabra*), cinnamoylechinaxanthol from *Echinacea* root; cimiphenol from black cohosh (*Cimicifuga racemosa*), rosemarinic acid from rosemary (*Rosmarinus officinalis*), lemon balm (*Melissa officinalis*) and common sage (*Salvia officinalis*) [77]. Isoquercitrin, which is a flavonoid compound, has been found to interfere with the entry of the virion into the target cells [78]. Curcumin, a bioactive compound in turmeric also prevents ZIKV attachment to cells [79]. Gossypol, a phenolic compound seen in cotton seeds, has anti-ZIKV activity by interacting with the envelope protein domain III of the virus [80]. F-6 and FAc-2 fractions abundant in cyclic diterpenes with aldehyde groups from *Dictyota menstrualis*, a brown seaweed in Brazil, have potent anti-viral activity against ZIKV [81]. Polyphenols such as delphinidin and epigallocatechin gallate, which are available in natural products such as wine and tea, exhibited antiviral activity against ZIKV in an in vitro model [82,83]. Berberine, an isoquinoline alkaloid seen in *Berberis vulgaris, as well as* Emodin, an anthraquinone derivative available in *Rheum palmatum*, *Polygonum multiflorum*, *Aloe vera*, and *Cassia obtusifolia* were found to have anti-viral activity against ZIKV [84]. A flavonoid compound called naringenin seen in citrus plants exhibits anti-ZIKV activity by binding to the protease domain of the NS2B-NS3 protein [85]. Further, anti-ZIKV activity of flavonoids has also been extensively reviewed elsewhere [86]. For example, 6-deoxyglucose-diphyllin, seen in *Justicia gendarussa* could prevent the facilitation of an acidic environment within the lysosome or endosome that allows the virus to fuse. Further, 6-deoxyglucose-diphyllin was protective against ZIKV infection both in cell culture as well as in an immunocompromised mice model [87]. Hippeastrine hydrobromide seen in *Lycoris radiate* was found to be protective against the neuronal damage caused by ZIKV along with having other anti-viral activity [88]. *Doratoxylon apetalum* plant extract, which is already known to have a protective role against oxidative stress in cells, also had anti-viral activity by preventing ZIKV entry into the cells [89]. 25-hydroxy cholesterol seen naturally in the hosts was found to have anti-viral activity and was able to prevent ZIKV associated clinical signs in both mice and macaque models. Similarly, 25-hydroxy cholesterol also inhibited ZIKV infection in human corticoid organs and microcephaly in newborn mice pups [90]. An omega-3 polyunsaturated fatty acid, docosahexaenoic acid (DHA) was found to have a protective effect against ZIKV-induced neuronal damage in a cell culture model [91]. Nutraceuticals investigated for anti-ZIKV activity are listed in Table 2.

### 3.1. Other Nutraceuticals against ZIKV Infection

Harringtonine is a natural alkaloid from plant genus, *Cephalotaxus* and has been shown to show antiviral activities against ZIKV [92]. Harringtonine was shown to inhibit early and late-stage infection and inhibits ZIKV binding, entry and replication of virus. Molecular analysis of harringtonine shows that it can interact with ZIKV envelope proteins and blocks viral binding and entry into the host cells [92]. In addition, palmatine, a plant protoberberine alkaloid was shown to inhibit ZIKV infection by blocking viral binding, entry and stability of ZIKV in vero cells [93]. Cinnamic acid, an organic acid isolated from cinnamon twigs was also shown to inhibit ZIKV replication in vero cells, hepatocytes (Huh7), A549 cells, in vitro and in mouse model of interferon receptor-deficient mice (Infagr-/-) [94]. Cinnamic acid greatly improved the survival of ZIKV infected mice. [94]. Indole alkaloids from the seeds of *T. Cymosa* was also shown to possess anti-ZIKV activity and are not cytotoxic to Vero cells and A549 cells [95]. Further studies are required to elucidate the detailed mechanisms of anti-ZIKV activity of alkaloids in humans. The role of nutraceuticals in blocking ZIKV replication are represented in Figure 1.

**Table 2:** List of novel nutrient molecules and mechanism of protection against ZIKV infection.

Nutrient Molecule	ZIKV Strain	Cells	Result	Mechanism of Protection	Ref.No.
Schinus terebinthifolia, Ethanolic fruits’peel extract (STPE) and whole fruits extract (STWFE)	MR766 (African Strain) or PE243 (EH) ZIKV strains with 1 MOI	HTR-8/SVneo cells	Potential early antiviral effect, inhibited ZIKV entry	Resveratrol (present in STWFE and STPE) prevents ZIKV replication and exhibit virucidal activity	[96,97]
Isoquercitrin	PF-25013-18 (2 MOI for A549), and ZIKV MR766^MC^, viral clone derived African strain MR766-NIID (1 MOI for A549, Huh-7 and 10 MOI for SHSY5Y)	A549, Huh-7, SH-SY5Y	Potential inhibitor of ZIKV infection in different human cells tested	Plays an anti-ZIKV activity and glycosylated moiety present in Isoquercitrin plays a vital role. Prevents the ZIKV internalization into the host cell (prevents viral entry)	[78]
Curcumin (Pretreatment)	HD78788 with 0.1, 1, and 1 MOI	HeLa, BHK-21, and Vero-E	Decreased ZIKV infection in a time and dose dependent manner	Interferes with the ZIKV envelope binding to the cell though viral RNA integrity was maintained	[79]
Gossypol, digitonin, and conessine	PAN2016, R116265, PAN2015, FLR, R103451, PRVABC59, PLCal_ZV, IbH 30656, mosquito strain MEX 2–81, and African strain (MR 766)	Vero E6 cells	Compared to conessine and digitonin, gossypol exhibited the strong inhibitory activity against 10 different ZIKV strains	Gossypol target EDIII of ZIKV and neutralize the infectionConessine and digitonin targets the host cell entry and ZIKV replication stages	[80]
Dictyota menstrualis (F-6 and FAc-2 fractions)	MR 766 with 0.01–1 MOI	Vero cells	Dose-dependent inhibition of ZIKV replication (>74%)	F-6 inhibits viral adsorptionFAc-2-strong virucidal potential	[81]
Polyphenols—Delphinidin (D) and Epigallocate-chin gallate (EGCG)	African MR766 and the American PA259459 with ~10^6^ PFU	Vero cells	D and EGCG shows virucidal effect which decreases the ZIKV infection The virucidal of D and EGCG was higher in MR766 compared to PA259459 strain	Inhibition of two different ZIKV strains (MR766 and PA259459) by D and EGCG was different, mainly by EGCG.This may be due to E protein which has different amino acid composition. MR766 lacks glycosylation motif at position 154 and 4 amino acid deletion, which are found in Asian strains of ZIKV	[82,83]
Berberine and emodin	Brazilian Zika virus strain isolated from a febrile patient in northeast Brazil with 10^6^ PFU/mL	Vero E6 cells	Induces virucidal effect and decreases the ZIKV infection: 160 µM of berberine decreases infectivity by 77.6%, whereas 40 µM of emodin decreases by 83.3%.	The compounds act on the ZIKV structure. Hydrodynamic radius of the ZIKV was reduced with the treatment of Berberine and emodin	[84]
Harringtonine	PRVABC59	African Green Monkey Kidney cells	Inhibits ZIKV entry, replication and virion release	Virucidal effects, prophylaxis activity	[92]
Palmatine	ATCC VR-1843	Vero cells	Prevents ZIKV binding and entry	Virucidal effects	[93]
Cinnamic acid	Asian ZIKV	Vero cells, Huh7, A549	Prevent ZIKV replication	Inihibit RdRp activity	[94]
Naringenin (NAR) Treatment after infection	Viruses isolated from serum of infected patients in South Brazil (2016) and Northeast (2015). Human A549 lung epithelial cells: ZIKV (ZV BR 2015/15261, ZV BR 2016/16288, ZV BR 2015/15098, ZIKV PE243, ZIKV MR766) with 0.1 MOI Human monocyte-derived dendritic cells: ZIKV (ZV BR 2015/15261) with 10 MOI		In vitro NAR was effective against distinct ZIKV lineages (Asian and African) and seems to act during the late phase of the viral life cycle	Acts on the ZIKV replication or viral assembly on the host cell. Computation analysis, predicts that interaction between NS2B-NS3 protein in ZIKV and naringenin plays a vital role for the anti-ZIKA activity	[85]
6-deoxyglucose-diphyllin (DGP)	HT1080, VERO, and CHME3 cells with ZIKV-MR766 and ZIKV-RVPs at ~1 MOI. CHME3 cells with PRVABC59, BeH819015, IBH30656, and DAK-ArD-41524 with 1, 0.2, 0.2 and 0.5 MOI, respectively		Inhibits both in vitro and in vivo ZIKV infection	Based on virological and cellular experiments: Prevents at binding stage of ZIKV to the host cell (fusion) thus preventing the viral contents entry to the cytosol. Mechanistic studies: Block the acidification in the host cell at the endosomal/lysosomal compartments which prevents ZIKV fusion with the cell membrane	[87]
Doratoxylum apetalum	A549, clinical isolate PF-25013-18 of ZIKV (ZIKV- PF13) with 2 MOI Huh7.5 cells, Brazilian strain (ZIKV-BR) with 2 MOI Recombinant Zika virus expressing the GFP reporter gene (ZIKV^GFP^)		Anti-ZIKV activity with non-cytotoxic concentration in human cell lines	Prevents internalization of ZIKV particles into the host cell, thus preventing the ZIKV entry into the cell and viral particle inactivation.	[89]
Docosahexaenoic acid (DHA)	SH-SY5Y, ZIKV_PE243_ with 10 MOI		DHA shows neuroprotective and anti-inflammatory potential	DHA restores the mitochondrial function and inhibits reactive species production with ZIKV infection	[91]
Polydatin (natural precursor of resveratrol and commonly found in grape, peanut etc.)	Computational based approach: Molecular docking of phytochemical compounds against NS5 or RdRp, RNA dependent RNA polymerase		Out of 5000 phytochemicals screened, Polydatin shows the best binding interaction with NS5 RNA dependent RNA polymerase active site with docking score −18.71 kcal/mol. Compared to sofosbuvir, Polydatin has more capacity for the receptor binding		[75]

### 3.2. Nutrition and ZIKV

The nutritional status of the host can contribute to the evolution of the viral disease by mutations contributing to virulence [98]. Likewise, the nutritional status of the host can also play a role in the vector-borne disease evolution [99]. Evidence of supportive nutritional therapy has been important in arboviral infections such as dengue, but there are very limited reports surrounding the topic of nutritional parameters in the context of ZIKV infection [100]. There is an interesting hypothesis which states that the neuronal damage associated with ZIKV could result from the retinoid compounds that have leaked from the liver tissue during ZIKV infection [101]. A study found an association between nutrition and motor function in children with palsy; this could also possibly affect the outcomes in infants affected with ZIKV infection [102]. A correlation between ZIKV infection and anemia has been seen in several cases, and in contrast, other reports show no evidence of anemia with ZIKV infection [103,104]. A study using a mice model revealed that protein malnutrition could be a risk factor in developing congenital Zika syndrome. The results of this study are possibly correlated to the fact that undernutrition is commonly seen in regions with major Zika outbreaks such as Brazil [105]. Another interesting entomology study found that blood meal containing ZIKV showed prominent infection in mosquitoes when compared to protein meal containing ZIKV fed mosquitoes [106]. A study showed that folic acid supplementation reduced ZIKV infection in a cell culture model associated with placental barrier and showed improved postnatal outcomes in fetuses from ZIKV infected pregnant mice [107]. Therefore, monitoring folic acid nutrient status in ZIKV prone endemic areas and enabling its supplementation could help to ameliorate the adverse effect on the fetus observed during ZIKV infection [107].

### 3.3. Immunological Response to ZIKV Infection

ZIKV can evade the innate immune responses generated by the host via suppression of type I interferon and the subsequent activation of interferon-stimulated genes via non-structural viral proteins such as NS5 and NS4A [44,108]. Interferon λ is known to protect against ZIKV infection in female mice in cases of sexual route of transmission [109]. A recent study shows that at least a 12-month interval between an initial Dengue virus infection confers an effective cellular immune response against a subsequent ZIKV infection [110]. There are also contrasting studies that report pre-existing antibodies against the Dengue virus aggravating ZIKV infection in human explant and mouse models [111,112]. However, there is so far no clinical evidence of enhancement of ZIKV in dengue sero-positive patients. Production of IgM, which is the first antibody to appear against the first encounter with a pathogen, is found to be affected in populations with pre-existing antibodies against flavivirus [113]. Pre-existing antibodies against ZIKV can also cause severe outcomes after subsequent dengue infection due to antibody-dependent enhancement (ADE) in humans [114,115]. 

In vivo and in vitro, Studies have shown that interferon λ could be protective against ZIKV infection in the placenta [116,117,118]. On the other end, a recent study has shown that placental alkaline phosphatase stabilized by binding immunoglobulin protein (Bip) aids ZIKV infection in placental cells [119]. ZIKV activates an inflammatory response following infection by inflammasome complex formation resulting in IL-1β release [120,121,122]. The placenta undergoes extensive inflammatory changes following ZIKV infection with upregulation of cytokines such as IFN-γ and TNF-α and chemokines such as RANTES (regulated on activation, normal T cell expressed and secreted) and VEGFR-2 (vascular endothelial growth factor receptor-2) [123,124]. Toll-like receptor-3 (TLR-3), which a pattern recognition receptor of the innate immune system that senses double-stranded RNA seen during ZIKV replication, is involved in activation of the inflammatory response particularly in astrocytes [125]. Similarly, babies with congenital Zika syndrome were found to be highly associated with single nuclear polymorphisms in TLR-3 or TNF-α (tumor necrosis factor-alpha) alleles [126]. These studies allude to the importance of the inflammatory response in the ZIKV-induced pathological manifestations in the host. Another important component that protects against ZIKV infection is a cell-mediated immune response and its associated neutralizing antibody generation which is crucial for immunity. Meanwhile, immunologically privileged parts of the body such as the gravid uterus might be vulnerable to ZIKV infection [127]. A sero-surveillance report in Fiji and French Polynesia that experienced ZIKV outbreaks in 2013–2014 currently displays a pattern wherein the younger population comprising of children still has neutralizing antibodies against ZIKV but the older population comprising of adults showed a decline in the neutralizing antibody titer [128].

### 3.4. ZIKV and Inflammation

ZIKV is known to affect various organs in the body from the eyes to the reproductive organs. ZIKV especially strains from the Asian lineage, are known to produce an inflammatory response in the body [129]. A study in chicken embryo livers showed that ZIKV from Asian lineage (isolated from China) elicited a very intense inflammatory response in comparison to dengue virus infection [130]. ZIKV infection in non-pregnant mice caused acute inflammation of the ovaries without any long-term effects on the overall reproducing ability [131]. An immunocompetent C57BL/6J mice model with intravenous challenge study also suggests that ZIKV induces an inflammatory environment in the blood-brain barrier. Additionally, ZIKV elicits a strong inflammatory response in human retinal epithelial cells and chemokine, CXCL10 was highly expressed following infection [132]. There is also evidence of placentitis in pregnant women following ZIKV infection [124,133]. Transcriptome analysis of human umbilical vein endothelial cells (HUVEC) showed upregulation of several cytokines, chemokines and matrix metalloproteinases on ZIKV infection [134]. There are also cases of ZIKV-induced meningitis, encephalitis and myelitis in certain patients [135]. In a recent study, IL-22 was attributed to inflammation of the brain in newborn mice pups infected with ZIKV [136]. Mice models suggest that ZIKV can cause orchitis and epididymitis via pro-inflammatory cytokines and chemokines [137]. A recent study showed that ZIKV induces IL-1β levels and IL-1 receptor antogonist therapy prevents placental inflammation and fetal neuroinflammation [138]. Overall, ZIKV infection elicits inflammatory response in the host with adverse fetal outcome. 

### 3.5. Cell Death in ZIKV Infection

ZIKV is known to initiate apoptosis, in which both intrinsic pathways and extrinsic pathways contribute to cell death in the neuronal progenitor cells, leading to microcephaly [139,140]. The extrinsic pathway of apoptosis is activated by cytokines and death ligands such as FasL while the activation of the intrinsic pathway is by cytochrome C released from damaged mitochondria. Both intrinsic and extrinsic pathways merge into a common pathway by activating effector caspases that trigger apoptosis also known as programmed cell death [141]. Also, activation of the necroptotic pathway via RIPK1/RIPK3 (Receptor-interacting serine/threonine-protein kinase 1/3) and Z-DNA-binding protein 1 (ZBP1) favors succinate dehydrogenase formation in neuronal cells which interfere with ZIKV replication [142]. Pyroptosis is also known to be associated with ZIKV infection by its activation of NLRP3 complex formation, which initiates caspase-1 activation [143,144]. A recent study highlights the mechanism of pyroptosis in neuronal progenitor cells following ZIKV infection [145]. ZIKV replicates, forming complexes inside the endoplasmic reticulum, resulting in large vacuoles in the cytoplasmic compartment of the cell and leads to paraptosis in human epithelial cell lines, human primary fibroblasts and astrocytes [146]. Necrotic cell death involves swelling of internal cellular organelles, which eventually rupture and are released outside the cell [147]. Necrotic lesions involving the brain are also observed in animal models including immunocompetent mice and non-human primates [148,149]. Autophagy, once considered as a cell survival pathway by recycling cellular cargoes via lysosomes to build new cellular components, under certain conditions can activate cell death directly or indirectly [150]. ZIKV causes extensive activation of autophagy by downregulating Akt-mTOR (Protein kinase B- Mammalian target of rapamycin) signaling pathways aiding in replication [151]. In contrast, another study showed that activation of the mTOR pathway inhibits autophagy and facilitates ZIKV replication [152]. Together, ZIKV infection induces apoptosis, necrosis, pyroptosis, paraptosis and autophagy dependent cell death pathways.

### 3.6. ZIKV and Placenta

Structure and function of the placenta: The placenta is a temporary organ that develops between the fetus and the mother and participates in nutrient transport, waste exchange and metabolism [153]. In humans, the fetal part of the placenta is composed of the placental disc, umbilical cord, amnion and chorion, whereas the maternal part from the endometrium of the uterus is the decidua [154]. The major cell type that predominates in the placenta is the trophoblast, which includes syncytiotrophoblasts, villous cytotrophoblasts, and extravillous trophoblasts that are characterized by a highly invasive nature, supported by the maternal decidual cells [155]. The placental functional units are called villi, formed by an outer layer of trophoblasts with a stromal core [156]. The placental villi participate in nutrient absorption for the growing fetus like the intestinal villi that absorb nutrients from digested food in the gastrointestinal tract [157,158]. Cytotrophoblasts are a layer of cells that cover the stromal core located between the basement membrane and syncytiotrophoblasts [159]. Extravillous trophoblasts are cells that migrate from the villi and are involved in uterine remodeling by forming trophoblast cell columns. Syncytiotrophoblasts are multinucleated cells covering the entire placental units, 2–3 cytotrophoblasts fusing to form syncytiotrophoblasts [160]. Both syncytiotrophoblasts and extravillous trophoblasts are differentiated from the cytotrophoblasts [161]. The stromal core of the placenta is richly supplied with blood vessels that originate from the mesenchymal stem cells and Hofbauer cells (placental macrophages) [161]. 

### 3.7. ZIKV Infection in the Placenta and Its Consequences

ZIKV has been demonstrated to replicate in the human placenta, including the Hofbauer cells and trophoblasts [162,163,164,165,166,167]. T cell immunoglobulin and mucin domain 1 (TIM1), Tyro3 and Axl (tyrosine-protein kinase receptors) are considered the cofactors for viral entry into cells. There is a considerable expression of TIM1 in cytotrophoblasts, fibroblasts, umbilical vein endothelial cells, Hofbauer cells and amniochorionic membranes of the placenta, whereas Tyro3 and Axl are variably expressed in these cells [168]. The first trimester of pregnancy was reported to be most susceptible to ZIKV infection [56,133,169,170], while some reports demonstrated that Congenital Zika Syndrome was also observed with ZIKV infection during the second and the third trimesters of pregnancy [171,172,173]. Placental enlargement is an early clinical feature noticed in ZIKV infected pregnancies [174]. Pregnant women who delivered babies with microcephaly typically exhibit clinical signs of ZIKV infection around the start of mid-gestation (8–16 weeks); this is when maternal blood circulation is well established via the placenta [175]. Breaches in the placental barrier could be detected in placental sections from ZIKV infected women [133]. A study using a cell culture model suggests that ZIKV can breach the placental barrier by disruption of tight junctions between the cells of the placenta. Further, ZIKV virions take a transcytosis route to enter the tightly regulated placental barrier and blood-brain barrier [176]. Another study using placenta samples from ZIKV infected women reported that there are ongoing changes in the tight junctions of the syncytiotrophoblasts with decrease in the claudin 4 expression that leads to potential breaches of the placental barrier [177]. ZIKV could also be transferred from the placenta to the fetus utilizing secretory autophagy [178]. 

ZIKV infection alters the lipid metabolism of placental cells by favoring lipid droplet deposition, which contributes to the ongoing inflammatory process coupled with mitochondrial dysfunction [124]. Another study reported the association of sphingolipids and deposition of ceramides with ZIKV replication particularly in neuronal progenitor cells [179]. A study in ZIKV infected women showed that the placental samples had an inflammatory state even without the actual presence of ZIKV virion and connects this to the involvement of a modulatory role of anti-inflammatory protein annexin 1 (ANXA1) as a result of ZIKV exposure to the placenta [180]. Further, the presence of non-neutralizing flavivirus antibodies was also shown to facilitate or enhance viral infection and spread to syncytiotrophoblasts via neonatal Fc gamma receptor (FcRn) [181]. Recombination-activating gene-1(RAG-1) knockout mice treated with interferon α/β receptor (IFNAR 1) antibody study shows that neutrophils and macrophages of the dam can play an important role in limiting ZIKV spread to the fetus [182]. A recent study in twins found that trophoblasts from a baby without congenital Zika syndrome had a differential activation of genes which contributed to its ability to mount a better immune response against the infection [183]. Expression of Insulin-like growth factor II (IGF2), which is necessary for the proper development of the baby, was found to be inhibited in placental samples from ZIKV infected women [184]. Researchers were even able to rescue ZIKV in vitro from mesenchymal stem cells derived from the placenta of a woman who had cleared the infection and delivered a baby negative for ZIKV infection alluding to ZIKV persistence [185]. 

In a normal pregnancy, monocytes are polarized to the M2 state to be compatible with the placenta and uterine environment and generate an anti-inflammatory or immunosuppressive state with potential suppression of type I interferon response, but monocytes are predominantly polarized to the M1 pro-inflammatory phenotype, resulting in adverse outcomes of pregnancy due to ZIKV (African strain) infection [186]. The CD14^+^CD16^+^ monocytes in the peripheral circulation are also affected by ZIKV infection, producing a 100-fold increase in the expression of CXCL12 and IL-6 in ZIKV infected women [187]. 

### 3.8. Animal Models of ZIKV Infection during Pregnancy

A study of ZIKV infection in pregnant rhesus macaques confirmed extensive uteroplacental pathology leading to decreased oxygen permeability. This model recapitulates the adverse outcomes noticed in humans such as reduced intrauterine growth and still-birth [188]. Similarly, immunocompetent wild-type male mice mated with immunocompromised (interferon α/β receptor knock-out (IFNAR -/-)) female mice resulting in heterozygous pups which are immunocompetent and exhibited extensive placental pathology and fetal damage following ZIKV infection [189,190]. Further extensive placental labyrinth apoptosis contributed to hypoxia in the heterozygous fetus resulting in early resorption [190]. Recently, ZIKV infection to humanized STAT2 knockin mice during pregnancy showed increased placental and fetal brain infection [191].

## 4. Endoplasmic Reticulum (ER) Stress in ZIKV Infection

The ER is an important subcellular organelle in a eukaryotic cell wherein the oxidizing environment and chaperones serve the purpose of protein folding, and it also serves as the site for steroid hormone and lipid synthesis [192]. Several conditions including hypoxia, nutrient deprivation and perturbations in the calcium homeostasis cause accumulation of misfolded proteins in the ER leading to ER stress. This in turn results in the activation of several interactive signaling pathways known as the unfolded protein response (UPR). During ER stress, cells can undergo two possible fates: cell survival and cell death. The UPR tries to remove the misfolded proteins in the ER using different pathways to help cell survival, but persistent or prolonged ER stress can overwhelm the protective mechanism that aids in cell survival, resulting in the activation of cell death pathways such as apoptosis [193]. 

### 4.1. The three Arms of the ER Stress Pathway

**Figure 2:**
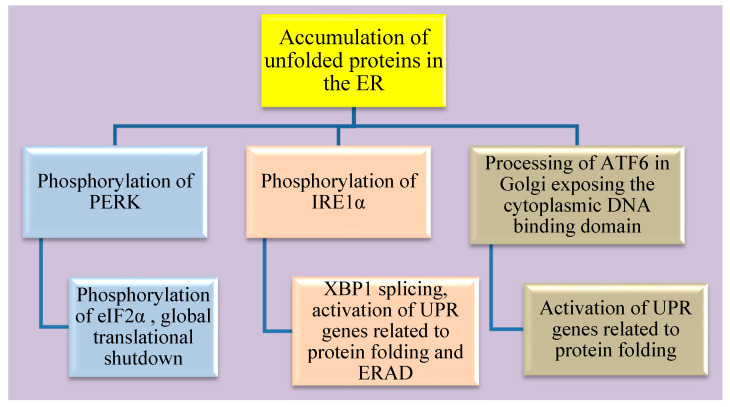
Schematic representation of the three arms of ER stress and its downstream targets.

There are three main arms of the UPR activation in cells with ER stress as shown in Figure 2. Usually, these sensors of unfolded proteins are at an inactive monomeric state with the association of Bip. It dissociates with a loss of homeostasis between folded and unfolded proteins leading to activation of ER stress sensors: (1) protein kinase RNA like endoplasmic reticulum kinase (PERK), (2) inositol requiring enzyme 1 alpha (IRE1α) and (3) activating transcription factor 6 (ATF6).

Flaviviruses are closely associated with ER stress as they replicate within the cellular membrane-bound organelles especially the ER. Accumulation of structural and non-structural proteins in the ER results in the formation of convoluted spherules which activate UPR. Apart from this, ZIKV also remodels the ER in terms of its protein and lipid content [194,195]. The shift from direct to indirect neurogenesis is carried out by decreasing UPR activity. Any dysregulation in this response could lead to microcephaly. ZIKV infection in neuronal progenitor cells activates PERK and IRE1α signaling pathways, suggesting the molecular mechanism behind the cause of microcephaly [196]. Further, ZIKV could indirectly activate UPR in response to ER stress by inducing cytokine and chemokine production in infected and non-infected neuronal cells. This also attracts the resident macrophages (microglial cells), amplifying the response [197]. ZIKV can also thwart the UPR mechanism in cells to counteract ER stress by downregulating Bip in A549 cells (human alveolar basal epithelial cell line) [198].

Additionally, there is cross-talk between the pathways of these three arms. Activation of the PERK pathway inhibits global protein synthesis via the phosphorylation of eukaryotic initiation factor 2 (eIf2α). ZIKV infected neuronal cells also show activation of the PERK pathway involving phosphorylation of eIf2α and activation of other targets such as ATF4, ATF3 and CHAC1(glutathione-specific γ-glutamylcyclotransferase 1) [196,199]. Activation of IRE1α which has endoribonuclease activity splices out a 26-nucleotide intron from X-box binding protein 1 (XBP1) resulting in a frameshift. This spliced XBP1 is now a transcription factor and can translocate to the nucleus for the upregulation of UPR-related genes that are involved in protein folding and endoplasmic reticulum-associated degradation pathways (ERAD) [200]. ZIKV is known to activate the IRE1α arm including XBP1 gene splicing, ER degradation-enhancing α-mannosidase-like 1 (EDEM-1) activation in neuronal cells [199]. Activation of IRE1α and XBP1 gene-splicing facilitates lipid droplet production via stearoyl coenzyme A desaturase 1 (SCD1) [201]. ER stress also induces the activation of ATF6 and is translocated from the ER to the Golgi apparatus where it is processed to expose its cytoplasmic DNA binding domain. This fragment of ATF6 processed in the Golgi apparatus acts by upregulating the expression of UPR related genes that aid in protein folding [202]. ATF6 is known to positively regulate XBP1 gene expression during UPR activation. Further, ATF6 pathway is activated in ZIKV infected neuronal cells via its nuclear translocation [199].

### 4.2. Cellular Fate of Sustained ER Stress

Infection, starvation or hypoxia triggers unfolded protein accumulation in cells resulting in the activation and cross talk among the three arms of ER stress leading to translation arrest, activation genes involved in protein folding and ERAD.

**Figure 3:**
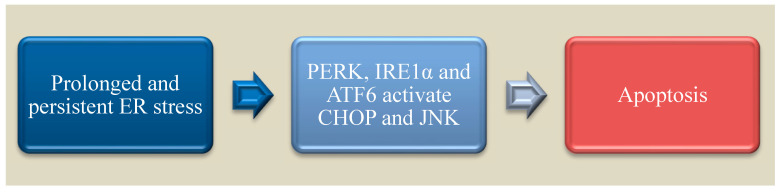
Schematic representation of pathways that lead the cell to progress from ER stress towards apoptosis.

Though cell survival is the main goal of signals generated through the three arms of ER stress, alternatively, persistent or sustained ER stress can activate apoptotic pathways as described in Figure 3. ER stress-induced apoptosis is regulated by C/EBP homologous protein (CHOP), c-Jun N-terminal kinase (JNK) and the B-cell lymphoma 2 (Bcl-2) family of proteins giving way to the activation of caspases [203]. All three arms can cause transcriptional activation of CHOP. CHOP acts mainly by the downregulation of anti-apoptotic activity of Bcl-2 and also acts by upregulating other targets such as growth arrest and DNA damage-inducible gene 34 (GADD34), endoplasmic reticulum oxidoreductase 1 alpha (ERO1α) and tribbles-related protein 3 (TRB3) [204,205,206]. Activated JNK phosphorylates Bcl-2 and BIM and results in the activation of bax and bak in the mitochondria, thereby initiating the intrinsic pathway of apoptosis [207]. CHOP activation is also linked to ER stress and apoptosis in ZIKV infected neuronal cells [199]. MAPK Kinases such as JNK and p38 also play a critical role in the infection of flavivirus like dengue [208]. Reticulophagy or ER-phagy cooperatively works with UPR to restore homeostasis in the ER membranes, but ZIKV can suppress this reticulophagy by reducing family with sequence similarity 134 member B (FAM134B) [209].

ER stress in cells over some time results in activation of CHOP and JNK which drives the cells to undergo apoptosis by the activation of terminal caspases. Altogether, ZIKV can infect the placenta resulting in vertical transmission to the fetus and the underlying molecular mechanism involves prolonged ER stress leading to apoptosis.

### 4.3. Palmitoleate

The need for safe treatment or control methods in ZIKV infected pregnant women to prevent adverse effects in the developing fetus paved a pathway to study the use of palmitoleate, a nutrient compound, as a potential therapeutic agent against ZIKV.

#### 4.3.1. Structure and Sources

Palmitoleate is an omega 7 monounsaturated fatty acid (16:1n-7) with a double bond in the seventh carbon atom counted from the methyl group with total 16 carbon atoms [210]. It is found predominantly in adipose tissue and blood [211,212]. Mammals can produce the cis form of palmitoleic acid synthesized from the saturated fatty acid palmitate via stearoyl-CoA desaturase 1 enzyme (SCD1) [213]. The trans-form of palmitoleic acid can be obtained from the consumption of dairy products and meat with fat content [213,214,215]. Next to oleate, palmitoleate is the second most abundant monounsaturated fatty acid in the body [215]. Plant sources of palmitoleate are macadamia nuts (*Macadamia integrifolia)*, seabuckthorn (*Hippophae rhamnoides),* Durian fruits (*Durio graveolens) and blue-green algae* [215,216]. Macadamia nuts alone contain 15–22% of palmitoleate occurring in its cis form [216,217,218,219]. 

#### 4.3.2. Lipokine Activity

Palmitoleate is a lipokine that can be released by fat tissue deposits, exerting functional roles in various organs [213]. Palmitoleate released from fat tissue lysis during endurance exercise regimes is known to cause cardiomyocyte hypertrophy [220]. Palmitoleate addition to bovine adipose tissue cultures promoted fatty acid oxidation and prevented lipogenesis [221]. Similarly, systemic administration of palmitoleate reduced intramuscular fat deposition and improved insulin sensitivity in obese sheep [222]. A macadamia nut-rich diet in humans has been shown to improve the lipid profile by decreasing low-density lipoprotein (LDL) [223,224]. Supplementation of palmitoleate along with western diet in LDL receptor knock-out mice showed a significant reduction in the size of atherosclerotic plaques in the heart when compared to mice supplemented with high oleic acid olive oil or control mice fed with western diet alone [225]. Further, palmitoleate can inhibit gap junction communication in vascular endothelial cells [226]. A cohort study involving people with different ethnic backgrounds in the USA found that high levels of circulating trans palmitoleate were correlated to a reduced risk of diabetes [214]. Another cohort study in non-diabetic individuals also found a correlation between high levels of circulating palmitoleate and improved insulin sensitivity, as the palmitoleic acid can reduce fatty acid-induced damage which affects the beta cells of the pancreas and the glucose metabolism [227].

#### 4.3.3. Positive Effects on Metabolic Health

Palmitoleate could change the polarization of macrophages from the inflammatory M1 macrophage seen in animals fed with an obesogenic diet to the anti-inflammatory M2 macrophage through activation of adenosine monophosphate-activated protein kinase (AMPK) [228]. The overall health benefits from palmitoleate supplementation in reducing inflammation, insulin resistance and preventing cardiovascular diseases could be attributed to the activation of AMPK supporting energy generation rather than its utilization [229]. Palmitoleate supplementation reduces the inflammation in high-fat diet-induced fatty liver conditions [230,231]. Palmitoleate was found to promote lipolysis via hormone-sensitive lipase (HSL) and adipose triglyceride lipase (ATGL) activity in adipose tissue through peroxisome proliferator-activated receptor alpha (PPARα) activation [232]. It was observed that the synthesis of palmitoleate was reduced in syncytiotrophoblasts isolated from obese mothers. This reduced palmitoleate production could aid in promoting an inflammatory environment and reduced insulin sensitivity in obese mothers [233]. In a rodent study, palmitoleate was also found to augment the wound healing process, which could be due to its anti-inflammatory property [234]. Palmitoleate was found to release satiety hormone cholecystokinin from the small intestine following oral supplementation in male rats [235]. Conversely, increased activity of the SCD1 enzyme that converts palmitic acid to palmitoleic acid results in insulin resistance, fatty liver and metabolic syndrome [236]. Another study in male physicians observed that increased palmitoleate levels in the red blood cell (RBC) correlated to an increased risk of cardiovascular disease [237]. When enveloped φ6 bacteriophages were treated with monounsaturated fatty acids such as palmitoleic acid and oleic acid, it resulted in inhibition of viral replication but saturated fatty acids like palmitic acid or myristic acid did not inhibit viral replication [238]. Overall, palmitoleate has several arrays of functional roles in the body.

#### 4.3.4. Palmitoleate Protects against ZIKV Infection

**Figure 4:**
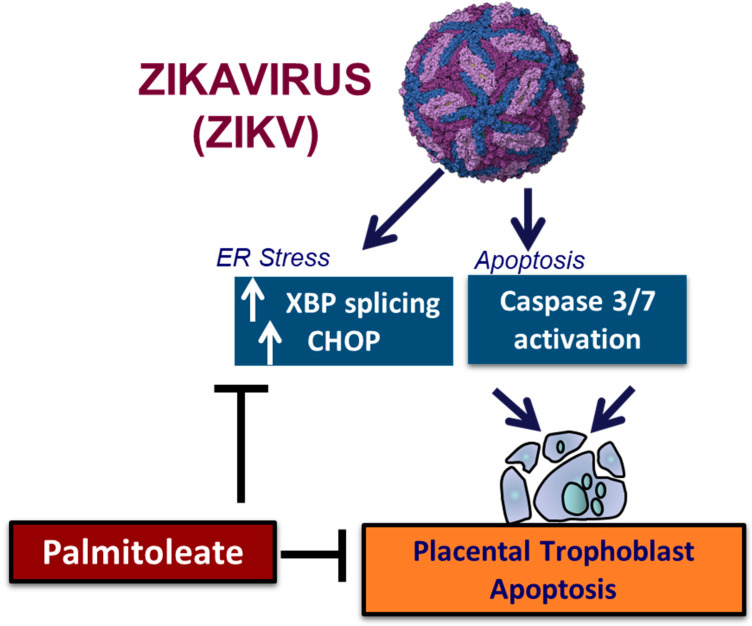
The schematic diagram represents palmitoleate protection against ZIKV-induced ER stress and apoptosis in placental trophoblasts.

Zika virus is known to cause apoptosis via sustained ER stress in the trophoblasts [167]. We have recently established that (1) palmitoleate, an omega 7 monounsaturated fatty acid significantly reduces ZIKV infection-induced trophoblast apoptosis; (2) treatment of palmitoleate interferes with ZIKV replication in trophoblasts; (3) palmitoleate treatment after ZIKV infection in trophoblasts downregulates the activation of ER stress markers that occur due to viral protein overload; and (4) palmitate, a saturated fatty acid with similar carbon structure to palmitoleate augments cell death in ZIKV-infected trophoblasts. Further, ZIKV infection in trophoblasts elicits ER stress via the upregulation of CHOP and XBP1 mRNA splicing which in turn activates apoptosis (Table 3). Supplementation of palmitoleate protects against ZIKV-induced ER stress and trophoblast apoptosis as described in Figure 4.

## 5. Summary

In summary, Zika virus (ZIKV) infection during pregnancy is associated with the development of Congenital Zika syndrome which involves birth defects such as intrauterine growth restriction (IUGR), ocular damage and microcephaly to the fetus. ZIKV infection to the placenta plays a crucial role in disease transmission from mother to fetus. We recently demonstrated that ZIKV infection induces endoplasmic reticulum (ER) stress- and mitogen activated protein kinase-dependent trophoblast apoptosis. We have also shown that palmitoleate protects ZIKV-induced trophoblast apoptosis. Further studies are required to test the protective role of palmitoleate against human ZIKV infection and in animal models such as using humanized STAT2 knock-in mice.

## Figures and Tables

**Table 1:** Zika viral proteins and their function.

Protein	Function
Envelope (E)	Host cell binding and membrane fusion [38]
Capsid	Viral protein surrounds nucleic acid [39]
Membrane protein	Proteolytic cleavage of a pre membrane protein from membrane protein in the Golgi apparatus results in the release of the virus [40]
NS1	RNA replication [41]
NS2A	Modulates different components of the virus during assembly [42]
NS2B	Cofactor of NS3 protease [43]
NS3	Protease and helicase domain for polyprotein possessing & nucleoside triphosphtase (NTPase)/RNA triphosphatase (RTPase) activities [43]
NS4A	Evasion of the innate immune response, associated with replication complex [44,45]
NS4B	Evasion of the innate immune response [46]
NS5	Methyl transferase (MTase) and RNA dependent RNA polymerase (RdRp) [47]

**Table 3:** ZIKV infection in placental trophoblast apoptosis involves activation of JNK and endoplasmic reticulum and Palmitoleate protects against ZIKV-induced ER stress and apoptosis in trophoblasts.

ZIKV Infection Induces ER Stress and Trophoblast Apoptosis [167]			
ZIKV Strain	Cells	Results	Apoptosis Mechanism
MR766 strain, recombinant MR766 strain, and PRVABC-59 strain with 0.1–1 MOI	HTR-8 (HTR-8/SVneo), JEG-3 and JAR	ZIKV infection induces ER stress and apoptosis in placental trophoblast.	*Extrinsic and Intrinsic Pathway:* -ZIKV increases caspase 3/7 activity and percent Apoptotic nuclear morphological changes -ZIKV induces caspase-depended apoptosis -*ER Stress markers:* Sustained ER stress results in Apoptosis.-increases CHOP mRNA and protein expression -increases P-IRE1α, spliced form of XBP1 mRNA, P-eIF2α -activation of JNK and MAPK *Critical mediator for apoptosis:* JNK and caspase activation acts as critical mediators for placental trophoblast apoptosis
Palmitoleate (PO) protects ZIKV infection-induced ER stress and apoptosis in trophoblasts [166]			
ZIKV strain	Cells	Results	PO protection mechanism
MR766 strain or recombinant MR766 or PRVABC59 with 0.1–1 MOI	HTR-8 (HTR-8/SVneo), JEG-3 and JAR	-PO decreases apoptotic nuclei % and caspase 3/7 activity -PO decreases CHOP mRNA expression level and spliced XBP1 mRNA -PO decreases viral envelope RNA copy no. and viral E protein expression -Palmitate treatment augments ZIKV-induced trophoblast apoptosis	*Possible mechanisms:* -Preventing ZIKV binding to the cell receptor -Lipid component of E protein -Preventing viral replication complex

## Data Availability

Not applicable.

## References

[1] Dick G.W., Kitchen S.F., Haddow A.J. (1952). Zika virus. I. Isolations and serological specificity. Trans. R. Soc. Trop. Med. Hyg..

[2] Dick G.W.A. (1952). Zika virus (II). Pathogenicity and physical properties. Trans. R. Soc. Trop. Med. Hyg..

[3] Simpson D.I.H. (1964). Zika virus infection in man. Trans. R. Soc. Trop. Med. Hyg..

[4] Smith D.E., Beckham J.D., Tyler K.L., Pastula D.M. (2016). Zika virus disease for neurologists. Neurol. Clin. Pract..

[5] Duffy M.R., Chen T.H., Hancock W.T., Powers A.M., Kool J.L., Lanciotti R.S., Pretrick M., Marfel M., Holzbauer S., Dubray C. (2009). Zika virus outbreak on Yap Island, Federated States of Micronesia. N. Engl. J. Med..

[6] Cao-Lormeau V.-M., Roche C., Teissier A., Robin E., Berry A.-L., Mallet H.-P., Sall A.A., Musso D. (2014). Zika virus, French polynesia, South pacific, 2013. Emerg. Infect. Dis..

[7] Oehler E., Watrin L., Larre P., Leparc-Goffart I., Lastere S., Valour F., Baudouin L., Mallet H., Musso D., Ghawche F. (2014). Zika virus infection complicated by Guillain-Barre syndrome--case report, French Polynesia, December 2013. Euro Surveill. Bull. Eur. Mal. Transm. = Eur. Commun. Dis. Bull..

[8] Musso D., Gubler D.J. (2016). Zika Virus. Clin. Microbiol. Rev..

[9] Kama M., Aubry M., Naivalu T., Vanhomwegen J., Mariteragi-Helle T., Teissier A., Paoaafaite T., Hue S., Hibberd M.L., Manuguerra J.C. (2019). Sustained Low-Level Transmission of Zika and Chikungunya Viruses after Emergence in the Fiji Islands. Emerg. Infect. Dis..

[10] Calvez E., Mousson L., Vazeille M., O’Connor O., Cao-Lormeau V.-M., Mathieu-Daudé F., Pocquet N., Failloux A.-B., Dupont-Rouzeyrol M. (2018). Zika virus outbreak in the Pacific: Vector competence of regional vectors. PLoS Negl. Trop. Dis..

[11] Delatorre E., Fernandez J., Bello G. (2018). Investigating the Role of Easter Island in Migration of Zika Virus from South Pacific to Americas. Emerg. Infect. Dis..

[12] Pettersson J.H.O., Eldholm V., Seligman S.J., Lundkvist Å., Falconar A.K., Gaunt M.W., Musso D., Nougairède A., Charrel R., Gould E.A. (2016). How Did Zika Virus Emerge in the Pacific Islands and Latin America?. mBio.

[13] Metsky H.C., Matranga C.B., Wohl S., Schaffner S.F., Freije C.A., Winnicki S.M., West K., Qu J., Baniecki M.L., Gladden-Young A. (2017). Zika virus evolution and spread in the Americas. Nature.

[14] Likos A., Griffin I., Bingham A.M., Stanek D., Fischer M., White S., Hamilton J., Eisenstein L., Atrubin D., Mulay P. (2016). Local Mosquito-Borne Transmission of Zika Virus—Miami-Dade and Broward Counties, Florida, June-August 2016. MMWR. Morb. Mortal. Wkly. Rep..

[15] Beaver J.T., Lelutiu N., Habib R., Skountzou I. (2018). Evolution of Two Major Zika Virus Lineages: Implications for Pathology, Immune Response, and Vaccine Development. Front. Immunol..

[16] Haddow A.D., Schuh A.J., Yasuda C.Y., Kasper M.R., Heang V., Huy R., Guzman H., Tesh R.B., Weaver S.C. (2012). Genetic characterization of Zika virus strains: Geographic expansion of the Asian lineage. PLoS Negl. Trop. Dis..

[17] Sheridan M.A., Balaraman V., Schust D.J., Ezashi T., Roberts R.M., Franz A.W.E. (2018). African and Asian strains of Zika virus differ in their ability to infect and lyse primitive human placental trophoblast. PLoS ONE.

[18] Enfissi A., Codrington J., Roosblad J., Kazanji M., Rousset D. (2016). Zika virus genome from the Americas. Lancet.

[19] Musso D. (2015). Zika Virus Transmission from French Polynesia to Brazil. Emerg. Infect. Dis..

[20] Li M.I., Wong P.S., Ng L.C., Tan C.H. (2012). Oral susceptibility of Singapore Aedes (Stegomyia) aegypti (Linnaeus) to Zika virus. PLoS Negl. Trop. Dis..

[21] Foy B.D., Kobylinski K.C., Chilson Foy J.L., Blitvich B.J., Travassos da Rosa A., Haddow A.D., Lanciotti R.S., Tesh R.B. (2011). Probable non-vector-borne transmission of Zika virus, Colorado, USA. Emerg. Infect. Dis..

[22] Mead P.S., Duggal N.K., Hook S.A., Delorey M., Fischer M., Olzenak McGuire D., Becksted H., Max R.J., Anishchenko M., Schwartz A.M. (2018). Zika Virus Shedding in Semen of Symptomatic Infected Men. N. Engl. J. Med..

[23] Müller J.A., Harms M., Krüger F., Groß R., Joas S., Hayn M., Dietz A.N., Lippold S., von Einem J., Schubert A. (2018). Semen inhibits Zika virus infection of cells and tissues from the anogenital region. Nat. Commun..

[24] Sharma A., Lal S.K. (2017). Zika Virus: Transmission, Detection, Control, and Prevention. Front. Microbiol..

[25] Elizondo-Quiroga D., Medina-Sánchez A., Sánchez-González J.M., Eckert K.A., Villalobos-Sánchez E., Navarro-Zúñiga A.R., Sánchez-Tejeda G., Correa-Morales F., González-Acosta C., Arias C.F. (2018). Zika Virus in Salivary Glands of Five Different Species of Wild-Caught Mosquitoes from Mexico. Sci. Rep..

[26] Mourya D.T., Gokhale M.D., Majumdar T.D., Yadav P.D., Kumar V., Mavale M.S. (2018). Experimental Zika virus infection in Aedes aegypti: Susceptibility, transmission & co-infection with dengue & chikungunya viruses. Indian J. Med. Res..

[27] Routhu N.K., Byrareddy S.N. (2017). Host-Virus Interaction of ZIKA Virus in Modulating Disease Pathogenesis. J. Neuroimmune Pharm..

[28] Newman C., Friedrich T.C., O’Connor D.H. (2017). Macaque monkeys in Zika virus research: 1947-present. Curr. Opin. Virol..

[29] Althouse B.M., Vasilakis N., Sall A.A., Diallo M., Weaver S.C., Hanley K.A. (2016). Potential for Zika Virus to Establish a Sylvatic Transmission Cycle in the Americas. PLoS Negl. Trop. Dis..

[30] Terzian A.C.B., Zini N., Sacchetto L., Rocha R.F., Parra M.C.P., Del Sarto J.L., Dias A.C.F., Coutinho F., Rayra J., da Silva R.A. (2018). Evidence of natural Zika virus infection in neotropical non-human primates in Brazil. Sci. Rep..

[31] Passi D., Sharma S., Dutta S.R., Ahmed M. (2017). Zika Virus Diseases—The New Face of an Ancient Enemy as Global Public Health Emergency (2016): Brief Review and Recent Updates. Int. J. Prev. Med..

[32] Sirohi D., Chen Z., Sun L., Klose T., Pierson T.C., Rossmann M.G., Kuhn R.J. (2016). The 3.8 Å resolution cryo-EM structure of Zika virus. Science.

[33] Basile K., Kok J., Dwyer D.E. (2017). Zika virus: What, where from and where to?. Pathology.

[34] Faye O., Freire C.C., Iamarino A., Faye O., de Oliveira J.V., Diallo M., Zanotto P.M., Sall A.A. (2014). Molecular evolution of Zika virus during its emergence in the 20(th) century. PLoS Negl. Trop. Dis..

[35] Lee I., Bos S., Li G., Wang S., Gadea G., Desprès P., Zhao R.Y. (2018). Probing Molecular Insights into Zika Virus⁻Host Interactions. Viruses.

[36] Lindenbach B.D., Rice C.M. (2003). Molecular biology of flaviviruses. Adv. Virus Res..

[37] Sironi M., Forni D., Clerici M., Cagliani R. (2016). Nonstructural Proteins Are Preferential Positive Selection Targets in Zika Virus and Related Flaviviruses. PLoS Negl. Trop. Dis..

[38] Chellasamy S.K., Devarajan S. (2019). Identification of Potential Lead Molecules for Zika Envelope Protein from In Silico Perspective. Avicenna J. Med. Biotechnol..

[39] Tan T.Y., Fibriansah G., Kostyuchenko V.A., Ng T.-S., Lim X.-X., Zhang S., Lim X.-N., Wang J., Shi J., Morais M.C. (2020). Capsid protein structure in Zika virus reveals the flavivirus assembly process. Nat. Commun..

[40] Nambala P., Su W.-C. (2018). Role of Zika Virus prM Protein in Viral Pathogenicity and Use in Vaccine Development. Front. Microbiol..

[41] Moreira-Soto A., de Souza Sampaio G., Pedroso C., Postigo-Hidalgo I., Berneck B.S., Ulbert S., Brites C., Netto E.M., Drexler J.F. (2020). Rapid decline of Zika virus NS1 antigen-specific antibody responses, northeastern Brazil. Virus Genes.

[42] Zhang X., Xie X., Zou J., Xia H., Shan C., Chen X., Shi P.Y. (2019). Genetic and biochemical characterizations of Zika virus NS2A protein. Emerg. Microbes Infect..

[43] Hilgenfeld R., Lei J., Zhang L., Hilgenfeld R., Vasudevan S.G. (2018). The Structure of the Zik.ka Virus Protease, NS2B/NS3pro. Dengue and Zika: Control and Antiviral Treatment Strategies.

[44] Hu Y., Dong X., He Z., Wu Y., Zhang S., Lin J., Yang Y., Chen J., An S., Yin Y. (2019). Zika virus antagonizes interferon response in patients and disrupts RIG-I–MAVS interaction through its CARD-TM domains. Cell Biosci..

[45] Rodriguez A.K., Muñoz A.L., Segura N.A., Rangel H.R., Bello F. (2019). Molecular characteristics and replication mechanism of dengue, zika and chikungunya arboviruses, and their treatments with natural extracts from plants: An updated review. EXCLI J..

[46] Ngueyen T.T.N., Kim S.J., Lee J.Y., Myoung J. (2019). Zika Virus Proteins NS2A and NS4A Are Major Antagonists that Reduce IFN-β Promoter Activity Induced by the MDA5/RIG-I Signaling Pathway. J. Microbiol. Biotechnol..

[47] Wang B., Thurmond S., Hai R., Song J. (2018). Structure and function of Zika virus NS5 protein: Perspectives for drug design. Cell. Mol. Life Sci. CMLS.

[48] Routhu N.K., Lehoux S.D., Rouse E.A., Bidokhti M.R.M., Giron L.B., Anzurez A., Reid S.P., Abdel-Mohsen M., Cummings R.D., Byrareddy S.N. (2019). Glycosylation of Zika Virus is Important in Host-Virus Interaction and Pathogenic Potential. Int. J. Mol. Sci..

[49] Šebera J., Dubankova A., Sychrovský V., Ruzek D., Boura E., Nencka R. (2018). The structural model of Zika virus RNA-dependent RNA polymerase in complex with RNA for rational design of novel nucleotide inhibitors. Sci. Rep..

[50] Sager G., Gabaglio S., Sztul E., Belov G.A. (2018). Role of Host Cell Secretory Machinery in Zika Virus Life Cycle. Viruses.

[51] Heinz F.X., Stiasny K. (2017). The Antigenic Structure of Zika Virus and Its Relation to Other Flaviviruses: Implications for Infection and Immunoprophylaxis. Microbiol. Mol. Biol. Rev..

[52] Pierson T.C., Diamond M.S. (2018). The emergence of Zika virus and its new clinical syndromes. Nature.

[53] Rawlinson W. (2016). Pregnancy, the placenta and Zika virus (ZIKV) infection. Microbiol. Aust..

[54] Moore C.A., Staples J.E., Dobyns W.B., Pessoa A., Ventura C.V., Fonseca E.B.d., Ribeiro E.M., Ventura L.O., Neto N.N., Arena J.F. (2017). Characterizing the Pattern of Anomalies in Congenital Zika Syndrome for Pediatric Clinicians. JAMA Pediatr..

[55] Oeser C., Ladhani S. (2019). An update on Zika Virus and Congenital Zika Syndrome. Paediatr. Child. Health.

[56] Lima G.P., Rozenbaum D., Pimentel C., Frota A.C.C., Vivacqua D., Machado E.S., Sztajnbok F., Abreu T., Soares R.A., Hofer C.B. (2019). Factors associated with the development of Congenital Zika Syndrome: A case-control study. BMC Infect. Dis..

[57] Barbi L., Coelho A.V.C., Alencar L.C.A., Crovella S. (2018). Prevalence of Guillain-Barré syndrome among Zika virus infected cases: A systematic review and meta-analysis. Braz. J. Infect. Dis. Off. Publ. Braz. Soc. Infect. Dis..

[58] St George K., Sohi I.S., Dufort E.M., Dean A.B., White J.L., Limberger R., Sommer J.N., Ostrowski S., Wong S.J., Backenson P.B. (2017). Zika Virus Testing Considerations: Lessons Learned from the First 80 Real-Time Reverse Transcription-PCR-Positive Cases Diagnosed in New York State. J. Clin. Microbiol..

[59] Singh R.K., Dhama K., Karthik K., Tiwari R., Khandia R., Munjal A., Iqbal H.M.N., Malik Y.S., Bueno-Marí R. (2018). Advances in Diagnosis, Surveillance, and Monitoring of Zika Virus: An Update. Front. Microbiol..

[60] Basile A.J., Ao J., Horiuchi K., Semenova V., Steward-Clark E., Schiffer J. (2019). Performance of InBios ZIKV Detect™ 2.0 IgM Capture ELISA in two reference laboratories compared to the original ZIKV Detect™ IgM Capture ELISA. J. Virol. Methods.

[61] Munoz-Jordan J.L. (2017). Diagnosis of Zika Virus Infections: Challenges and Opportunities. J. Infect. Dis..

[62] Peters R., Stevenson M. (2019). Zika virus diagnosis: Challenges and solutions. Clin. Microbiol. Infect..

[63] Atif M., Azeem M., Sarwar M.R., Bashir A. (2016). Zika virus disease: A current review of the literature. Infection.

[64] Van Rompay K.K.A., Keesler R.I., Ardeshir A., Watanabe J., Usachenko J., Singapuri A., Cruzen C., Bliss-Moreau E., Murphy A.M., Yee J.L. (2019). DNA vaccination before conception protects Zika virus–exposed pregnant macaques against prolonged viremia and improves fetal outcomes. Sci. Transl. Med..

[65] da Silva S., Oliveira Silva Martins D., Jardim A.C.G. (2018). A Review of the Ongoing Research on Zika Virus Treatment. Viruses.

[66] Singh R.K., Dhama K., Khandia R., Munjal A., Karthik K., Tiwari R., Chakraborty S., Malik Y.S., Bueno-Marí R. (2018). Prevention and Control Strategies to Counter Zika Virus, a Special Focus on Intervention Approaches against Vector Mosquitoes-Current Updates. Front. Microbiol..

[67] Igbinosa I.I., Rabe I.B., Oduyebo T., Rasmussen S.A. (2017). Zika Virus: Common Questions and Answers. Am. Fam. Physician.

[68] Meghani Z., Boëte C. (2018). Genetically engineered mosquitoes, Zika and other arboviruses, community engagement, costs, and patents: Ethical issues. PLoS Negl. Trop. Dis..

[69] Poland G.A., Ovsyannikova I.G., Kennedy R.B. (2019). Zika Vaccine Development: Current Status. Mayo Clin. Proc..

[70] das Neves Almeida R., Racine T., Magalhães K.G., Kobinger G.P. (2018). Zika Virus Vaccines: Challenges and Perspectives. Vaccines.

[71] Pattnaik A., Sahoo B.R., Pattnaik A.K. (2020). Current Status of Zika Virus Vaccines: Successes and Challenges. Vaccines.

[72] Devillers J. (2018). Repurposing drugs for use against Zika virus infection. SAR QSAR Environ. Res..

[73] Gardinali N.R., Marchevsky R.S., Oliveira J.M., Pelajo-Machado M., Kugelmeier T., Castro M.P., Silva A.C.A., Pinto D.P., Fonseca L.B., Vilhena L.S. (2020). Sofosbuvir shows a protective effect against vertical transmission of Zika virus and the associated congenital syndrome in rhesus monkeys. Antivir. Res..

[74] Mesci P., Macia A., Moore S.M., Shiryaev S.A., Pinto A., Huang C.-T., Tejwani L., Fernandes I.R., Suarez N.A., Kolar M.J. (2018). Blocking Zika virus vertical transmission. Sci. Rep..

[75] Rehman A., Ashfaq U.A., Javed M.R., Shahid F., Noor F., Aslam S. (2022). The Screening of Phytochemicals Against NS5 Polymerase to Treat Zika Virus Infection: Integrated Computational Based Approach. Comb. Chem. High Throughput Screen.

[76] Souyoul S.A., Saussy K.P., Lupo M.P. (2018). Nutraceuticals: A Review. Derm. Ther..

[77] Byler K.G., Ogungbe I.V., Setzer W.N. (2016). In-silico screening for anti-Zika virus phytochemicals. J. Mol. Graph. Model..

[78] Gaudry A., Bos S., Viranaicken W., Roche M., Krejbich-Trotot P., Gadea G., Desprès P., El-Kalamouni C. (2018). The Flavonoid Isoquercitrin Precludes Initiation of Zika Virus Infection in Human Cells. Int. J. Mol. Sci..

[79] Mounce B.C., Cesaro T., Carrau L., Vallet T., Vignuzzi M. (2017). Curcumin inhibits Zika and chikungunya virus infection by inhibiting cell binding. Antivir. Res..

[80] Gao Y., Tai W., Wang N., Li X., Jiang S., Debnath A.K., Du L., Chen S. (2019). Identification of Novel Natural Products as Effective and Broad-Spectrum Anti-Zika Virus Inhibitors. Viruses.

[81] Cirne-Santos C.C., Barros C.d.S., Gomes M.W.L., Gomes R., Cavalcanti D.N., Obando J.M.C., Ramos C.J.B., Villaça R.C., Teixeira V.L., Paixão I.C.N.d.P. (2019). In Vitro Antiviral Activity Against Zika Virus From a Natural Product of the Brazilian Brown Seaweed Dictyota menstrualis. Nat. Prod. Commun..

[82] Vazquez-Calvo A., de Oya N.J., Martin-Acebes M.A., Garcia-Moruno E., Saiz J.C. (2017). Antiviral Properties of the Natural Polyphenols Delphinidin and Epigallocatechin Gallate against the Flaviviruses West Nile Virus, Zika Virus, and Dengue Virus. Front. Microbiol..

[83] Fong Y.D., Chu J.J.H. (2022). Natural products as Zika antivirals. Med. Res. Rev..

[84] Batista M.N., Braga A.C.S., Campos G.R.F., Souza M.M., Matos R.P.A.d., Lopes T.Z., Candido N.M., Lima M.L.D., Machado F.C., Andrade S.T.Q.d. (2019). Natural Products Isolated from Oriental Medicinal Herbs Inactivate Zika Virus. Viruses.

[85] Cataneo A.H.D., Kuczera D., Koishi A.C., Zanluca C., Silveira G.F., Arruda T.B.d., Suzukawa A.A., Bortot L.O., Dias-Baruffi M., Verri W.A. (2019). The citrus flavonoid naringenin impairs the in vitro infection of human cells by Zika virus. Sci. Rep..

[86] Cataneo A.H.D., Avila E.P., Mendes L.A.O., de Oliveira V.G., Ferraz C.R., de Almeida M.V., Frabasile S., Duarte Dos Santos C.N., Verri W.A., Bordignon J. (2021). Flavonoids as Molecules With Anti-Zika virus Activity. Front. Microbiol..

[87] Martinez-Lopez A., Persaud M., Chavez M.P., Zhang H., Rong L., Liu S., Wang T.T., Sarafianos S.G., Diaz-Griffero F. (2019). Glycosylated diphyllin as a broad-spectrum antiviral agent against Zika virus. EBioMedicine.

[88] Zhou T., Tan L., Cederquist G.Y., Fan Y., Hartley B.J., Mukherjee S., Tomishima M., Brennand K.J., Zhang Q., Schwartz R.E. (2017). High-Content Screening in hPSC-Neural Progenitors Identifies Drug Candidates that Inhibit Zika Virus Infection in Fetal-like Organoids and Adult Brain. Cell Stem Cell.

[89] Haddad J.G., Koishi A.C., Gaudry A., Nunes Duarte Dos Santos C., Viranaicken W., Desprès P., El Kalamouni C. (2019). Doratoxylon apetalum, an Indigenous Medicinal Plant from Mascarene Islands, Is a Potent Inhibitor of Zika and Dengue Virus Infection in Human Cells. Int. J. Mol. Sci..

[90] Li C., Deng Y.-Q., Wang S., Ma F., Aliyari R., Huang X.-Y., Zhang N.-N., Watanabe M., Dong H.-L., Liu P. (2017). 25-Hydroxycholesterol Protects Host against Zika Virus Infection and Its Associated Microcephaly in a Mouse Model. Immunity.

[91] Braz-De-Melo H.A., Pasquarelli-do-Nascimento G., Corrêa R., das Neves Almeida R., de Oliveira Santos I., Prado P.S., Picolo V., de Bem A.F., Pizato N., Magalhães K.G. (2019). Potential neuroprotective and anti-inflammatory effects provided by omega-3 (DHA) against Zika virus infection in human SH-SY5Y cells. Sci. Rep..

[92] Lai Z.Z., Ho Y.J., Lu J.W. (2020). Harringtonine Inhibits Zika Virus Infection through Multiple Mechanisms. Molecules.

[93] Ho Y.J., Lu J.W., Huang Y.L., Lai Z.Z. (2019). Palmatine inhibits Zika virus infection by disrupting virus binding, entry, and stability. Biochem. Biophys. Res. Commun..

[94] Chen Y., Li Z., Pan P., Lao Z., Xu J., Li Z., Zhan S., Liu X., Wu Y., Wang W. (2021). Cinnamic acid inhibits Zika virus by inhibiting RdRp activity. Antivir. Res..

[95] Monsalve-Escudero L.M., Loaiza-Cano V., Pajaro-Gonzalez Y., Oliveros-Diaz A.F., Diaz-Castillo F., Quinones W., Robledo S., Martinez-Gutierrez M. (2021). Indole alkaloids inhibit zika and chikungunya virus infection in different cell lines. BMC Complement. Med. Ther..

[96] Mohd A., Zainal N., Tan K.K., AbuBakar S. (2019). Resveratrol affects Zika virus replication in vitro. Sci. Rep..

[97] Oliveira M.B.S., Valentim I.B., Rocha T.S., Santos J.C., Pires K.S.N., Tanabe E.L.L., Borbely K.S.C., Borbely A.U., Goulart M.O.F. (2020). Schinus terebenthifolius Raddi extracts: From sunscreen activity toward protection of the placenta to Zika virus infection, new uses for a well-known medicinal plant. Ind. Crops Prod..

[98] Beck M.A., Handy J., Levander O.A. (2004). Host nutritional status: The neglected virulence factor. Trends Microbiol..

[99] Weger-Lucarelli J., Auerswald H., Vignuzzi M., Dussart P., Karlsson E.A. (2018). Taking a bite out of nutrition and arbovirus infection. PLoS Negl. Trop. Dis..

[100] Wiwanitkit V. (2017). Nutritional Disorder in Zika Virus Infection. Int. J. Nutr. Disord. Ther..

[101] Mawson A.R. (2016). Pathogenesis of Zika Virus-Associated Embryopathy. BioResearch Open Access.

[102] Leandro C.G. (2016). Nutritional status and gross motor function in children with cerebral palsy, and implications for Zika virus infection. Dev. Med. Child. Neurol..

[103] Wiwanitkit S., Wiwanitkit V. (2016). Zika virus infection: No anemia. Ann. Trop. Med. Public Health.

[104] Schaub B., Vouga M., Najioullah F., Gueneret M., Monthieux A., Harte C., Muller F., Jolivet E., Adenet C., Dreux S. (2017). Analysis of blood from Zika virus-infected fetuses: A prospective case series. Lancet Infect. Dis..

[105] Barbeito-Andrés J., Pezzuto P., Higa L.M., Dias A.A., Vasconcelos J.M., Santos T.M.P., Ferreira J.C.C.G., Ferreira R.O., Dutra F.F., Rossi A.D. (2020). Congenital Zika syndrome is associated with maternal protein malnutrition. Sci. Adv..

[106] Huang Y.-J.S., Lyons A.C., Hsu W.-W., Park S.L., Higgs S., Vanlandingham D.L. (2017). Differential outcomes of Zika virus infection in Aedes aegypti orally challenged with infectious blood meals and infectious protein meals. PLoS ONE.

[107] Simanjuntak Y., Ko H.Y., Lee Y.L., Yu G.Y., Lin Y.L. (2020). Preventive effects of folic acid on Zika virus-associated poor pregnancy outcomes in immunocompromised mice. PLoS Pathog..

[108] Kumar A., Hou S., Airo A.M., Limonta D., Mancinelli V., Branton W., Power C., Hobman T.C. (2016). Zika virus inhibits type-I interferon production and downstream signaling. EMBO Rep..

[109] Caine E.A., Scheaffer S.M., Arora N., Zaitsev K., Artyomov M.N., Coyne C.B., Moley K.H., Diamond M.S. (2019). Interferon lambda protects the female reproductive tract against Zika virus infection. Nat. Commun..

[110] Serrano-Collazo C., Pérez-Guzmán E.X., Pantoja P., Hassert M.A., Rodríguez I.V., Giavedoni L., Hodara V., Parodi L., Cruz L., Arana T. (2020). Effective control of early Zika virus replication by Dengue immunity is associated to the length of time between the 2 infections but not mediated by antibodies. PLoS Negl. Trop. Dis..

[111] Castanha P.M.S., Erdos G., Watkins S.C., Falo L.D., Marques E.T.A., Barratt-Boyes S.M. (2020). Reciprocal immune enhancement of dengue and Zika virus infection in human skin. JCI Insight.

[112] Brown J.A., Singh G., Acklin J.A., Lee S., Duehr J.E., Chokola A.N., Frere J.J., Hoffman K.W., Foster G.A., Krysztof D. (2019). Dengue Virus Immunity Increases Zika Virus-Induced Damage during Pregnancy. Immunity.

[113] Malafa S., Medits I., Aberle J.H., Aberle S.W., Haslwanter D., Tsouchnikas G., Wölfel S., Huber K.L., Percivalle E., Cherpillod P. (2020). Impact of flavivirus vaccine-induced immunity on primary Zika virus antibody response in humans. PLoS Negl. Trop. Dis..

[114] Tang B., Xiao Y., Sander B., Kulkarni M.A., Radam-Lac Research T., Wu J. (2020). Modelling the impact of antibody-dependent enhancement on disease severity of Zika virus and de.engue virus sequential and co-infection. R Soc. Open Sci..

[115] Martín-Acebes M.A., Saiz J.-C., Jiménez de Oya N. (2020). Dengue Virus Strikes Back: Increased Future Risk of Severe Dengue Disease in Humans as a Result of Previous Exposure to Zika Virus. J. Clin. Med..

[116] Jagger B.W., Miner J.J., Cao B., Arora N., Smith A.M., Kovacs A., Mysorekar I.U., Coyne C.B., Diamond M.S. (2017). Gestational Stage and IFN-λ Signaling Regulate ZIKV Infection In Utero. Cell Host Microbe.

[117] Casazza R.L., Lazear H.M. (2020). Maternal interferon lambda signaling limits transplacental transmission and mediates fetal pathology during congenital Zika virus infection in mice. J. Immunol..

[118] Bayer A., Lennemann N.J., Ouyang Y., Bramley J.C., Morosky S., De Azeved Marques E.T., Cherry S., Sadovsky Y., Coyne C.B. (2016). Type III Interferons Produced by Human Placental Trophoblasts Confer Protection against Zika Virus Infection. Cell Host Microbe.

[119] Chen J., Chen Z., Liu M., Qiu T., Feng D., Zhao C., Zhang S., Zhang X., Xu J. (2020). Placental Alkaline Phosphatase Promotes Zika Virus Replication by Stabilizing Viral Proteins through BIP. mBio.

[120] Wang W., Li G., De W., Luo Z., Pan P., Tian M., Wang Y., Xiao F., Li A., Wu K. (2018). Zika virus infection induces host inflammatory responses by facilitating NLRP3 inflammasome assembly and interleukin-1β secretion. Nat. Commun..

[121] Clé M., Desmetz C., Barthelemy J., Martin M.-F., Constant O., Maarifi G., Foulongne V., Bolloré K., Glasson Y., De Bock F. (2020). Zika Virus Infection Promotes Local Inflammation, Cell Adhesion Molecule Upregulation, and Leukocyte Recruitment at the Blood-Brain Barrier. mBio.

[122] Zheng Y., Liu Q., Wu Y., Ma L., Zhang Z., Liu T., Jin S., She Y., Li Y.-P., Cui J. (2018). Zika virus elicits inflammation to evade antiviral response by cleaving cGAS via NS1-caspase-1 axis. EMBO J..

[123] Rabelo K., de Souza L.J., Salomão N.G., Machado L.N., Pereira P.G., Portari E.A., Basílio-de-Oliveira R., dos Santos F.B., Neves L.D., Morgade L.F. (2020). Zika Induces Human Placental Damage and Inflammation. Front. Immunol..

[124] Chen Q., Gouilly J., Ferrat Y.J., Espino A., Glaziou Q., Cartron G., El Costa H., Al-Daccak R., Jabrane-Ferrat N. (2020). Metabolic reprogramming by Zika virus provokes inflammation in human placenta. Nat. Commun..

[125] Ojha C.R., Rodriguez M., Karuppan M.K.M., Lapierre J., Kashanchi F., El-Hage N. (2019). Toll-like receptor 3 regulates Zika virus infection and associated host inflammatory response in primary human astrocytes. PLoS ONE.

[126] Santos C.N.O., Ribeiro D.R., Cardoso Alves J., Cazzaniga R.A., Magalhães L.S., de Souza M.S.F., Fonseca A.B.L., Bispo A.J.B., Porto R.L.S., Santos C.A.d. (2019). Association Between Zika Virus Microcephaly in Newborns With the rs3775291 Variant in Toll-Like Receptor 3 and rs1799964 Variant at Tumor Necrosis Factor-α Gene. J. Infect. Dis..

[127] Winkler C.W., Myers L.M., Woods T.A., Messer R.J., Carmody A.B., McNally K.L., Scott D.P., Hasenkrug K.J., Best S.M., Peterson K.E. (2017). Adaptive Immune Responses to Zika Virus Are Important for Controlling Virus Infection and Preventing Infection in Brain and Testes. J. Immunol..

[128] Henderson A.D., Aubry M., Kama M., Vanhomwegen J., Teissier A., Mariteragi-Helle T., Paoaafaite T., Teissier Y., Manuguerra J.C., Edmunds J. (2020). Zika seroprevalence declines and neutralizing antibodies wane in adults following outbreaks in French Polynesia and Fiji. eLife.

[129] Colavita F., Bordoni V., Caglioti C., Biava M., Castilletti C., Bordi L., Quartu S., Iannetta M., Ippolito G., Agrati C. (2018). ZIKV Infection Induces an Inflammatory Response but Fails to Activate Types I, II, and III IFN Response in Human PBMC. Mediat. Inflamm..

[130] Zhang Z., Sun M., Deng J., Yu J., Yang X., Zhao W., Chen G., Wang P. (2019). Zika Virus Induced More Severe Inflammatory Response Than Dengue Virus in Chicken Embryonic Livers. Front. Microbiol..

[131] Caine E.A., Scheaffer S.M., Broughton D.E., Salazar V., Govero J., Poddar S., Osula A., Halabi J., Skaznik-Wikiel M.E., Diamond M.S. (2019). Zika Virus Causes Acute Infection and Inflammation in the Ovary of Mice Without Apparent Defects in Fertility. J. Infect. Dis..

[132] Simonin Y., Erkilic N., Damodar K., Clé M., Desmetz C., Bolloré K., Taleb M., Torriano S., Barthelemy J., Dubois G. (2019). Zika virus induces strong inflammatory responses and impairs homeostasis and function of the human retinal pigment epithelium. EBioMedicine.

[133] Noronha L.d., Zanluca C., Azevedo M.L.V., Luz K.G., Santos C.N.D.D. (2016). Zika virus damages the human placental barrier and presents marked fetal neurotropism. Mem. Inst. Oswaldo Cruz.

[134] Khaiboullina S., Uppal T., Kletenkov K., St. Jeor S.C., Garanina E., Rizvanov A., Verma S.C. (2019). Transcriptome Profiling Reveals Pro-Inflammatory Cytokines and Matrix Metalloproteinase Activation in Zika Virus Infected Human Umbilical Vein Endothelial Cells. Front. Pharmacol..

[135] Leonhard S.E., Lant S., Jacobs B.C., Wilder-Smith A., Ferreira M.L.B., Solomon T., Willison H.J. (2018). Zika virus infection in the returning traveller: What every neurologist should know. Pract. Neurol..

[136] Liang Y., Yi P., Ru W., Jie Z., Wang H., Ghanayem T., Wang X., Alamer E., Liu J., Hu H. (2020). IL-22 hinders antiviral T cell responses and exacerbates ZIKV encephalitis in immunocompetent neonatal mice. J. Neuroinflammation.

[137] Ma W., Li S., Ma S., Jia L., Zhang F., Zhang Y., Zhang J., Wong G., Zhang S., Lu X. (2016). Zika Virus Causes Testis Damage and Leads to Male Infertility in Mice. Cell.

[138] Lei J., Vermillion M.S., Jia B., Xie H., Xie L., McLane M.W., Sheffield J.S., Pekosz A., Brown A., Klein S.L. (2019). IL-1 receptor antagonist therapy mitigates placental dysfunction and perinatal injury following Zika virus infection. JCI Insight.

[139] Souza B.S.F., Sampaio G.L.A., Pereira C.S., Campos G.S., Sardi S.I., Freitas L.A.R., Figueira C.P., Paredes B.D., Nonaka C.K.V., Azevedo C.M. (2016). Zika virus infection induces mitosis abnormalities and apoptotic cell death of human neural progenitor cells. Sci. Rep..

[140] Solomon I.H., Milner D.A., Folkerth R.D. (2016). Neuropathology of Zika Virus Infection. J. Neuroinfectious Dis..

[141] de Sousa J.R., Azevedo R.d.S.d.S., Quaresma J.A.S., Vasconcelos P.F.d.C. (2019). Cell Death And Zika Virus: An Integrated Network Of The Mechanisms Of Cell Injury. Infect. Drug Resist..

[142] Daniels B.P., Kofman S.B., Smith J.R., Norris G.T., Snyder A.G., Kolb J.P., Gao X., Locasale J.W., Martinez J., Gale M. (2019). The Nucleotide Sensor ZBP1 and Kinase RIPK3 Induce the Enzyme IRG1 to Promote an Antiviral Metabolic State in Neurons. Immunity.

[143] de Sousa J.R., Azevedo R., Martins Filho A.J., de Araujo M.T.F., Cruz E., Vasconcelos B.C.B., Cruz A.C.R., de Oliveira C.S., Martins L.C., Vasconcelos B.H.B. (2018). In situ inflammasome activation results in severe damage to the central nervous system in fatal Zika virus microcephaly cases. Cytokine.

[144] He Z., Chen J., Zhu X., An S., Dong X., Yu J., Zhang S., Wu Y., Li G., Zhang Y. (2018). NLRP3 Inflammasome Activation Mediates Zika Virus-Associated Inflammation. J. Infect. Dis..

[145] He Z., An S., Chen J., Zhang S., Tan C., Yu J., Ye H., Wu Y., Yuan J., Wu J. (2020). Neural progenitor cell pyroptosis contributes to Zika virus-induced brain atrophy and represents a therapeutic target. Proc. Natl. Acad. Sci. USA.

[146] Monel B., Compton A.A., Bruel T., Amraoui S., Burlaud-Gaillard J., Roy N., Guivel-Benhassine F., Porrot F., Genin P., Meertens L. (2017). Zika virus induces massive cytoplasmic vacuolization and paraptosis-like death in infected cells. EMBO J..

[147] Golstein P., Kroemer G. (2007). Cell death by necrosis: Towards a molecular definition. Trends Biochem. Sci..

[148] Figueiredo C.P., Barros-Aragão F.G.Q., Neris R.L.S., Frost P.S., Soares C., Souza I.N.O., Zeidler J.D., Zamberlan D.C., de Sousa V.L., Souza A.S. (2019). Zika virus replicates in adult human brain tissue and impairs synapses and memory in mice. Nat. Commun..

[149] Martinot A.J., Abbink P., Afacan O., Prohl A.K., Bronson R., Hecht J.L., Borducchi E.N., Larocca R.A., Peterson R.L., Rinaldi W. (2018). Fetal Neuropathology in Zika Virus-Infected Pregnant Female Rhesus Monkeys. Cell.

[150] Noguchi M., Hirata N., Tanaka T., Suizu F., Nakajima H., Chiorini J.A. (2020). Autophagy as a modulator of cell death machinery. Cell Death Dis..

[151] Gratton R., Agrelli A., Tricarico P.M., Brandão L., Crovella S. (2019). Autophagy in Zika Virus Infection: A Possible Therapeutic Target to Counteract Viral Replication. Int. J. Mol. Sci..

[152] Sahoo B.R., Pattnaik A., Annamalai A.S., Franco R., Pattnaik A.K. (2020). Mechanistic Target of Rapamycin Signaling Activation Antagonizes Autophagy To Facilitate Zika Virus Replication. J. Virol..

[153] Maltepe E., Fisher S.J. (2015). Placenta: The Forgotten Organ. Annu. Rev. Cell Dev. Biol..

[154] Caruso M., Evangelista M., Parolini O. (2012). Human term placental cells: Phenotype, properties and new avenues in regenerative medicine. Int. J. Mol. Cell Med..

[155] Turco M.Y., Moffett A. (2019). Development of the human placenta. Development.

[156] Gude N.M., Roberts C.T., Kalionis B., King R.G. (2004). Growth and function of the normal human placenta. Thromb. Res..

[157] Fuchs R., Ellinger I. (2004). Endocytic and Transcytotic Processes in Villous Syncytiotrophoblast: Role in Nutrient Transport to the Human Fetus. Traffic.

[158] Ensari A., Marsh M.N. (2018). Exploring the villus. Gastroenterol. Hepatol. Bed Bench.

[159] Parolini O., Alviano F., Bagnara G.P., Bilic G., Bühring H.-J., Evangelista M., Hennerbichler S., Liu B., Magatti M., Mao N. (2008). Concise Review: Isolation and Characterization of Cells from Human Term Placenta: Outcome of the First International Workshop on Placenta Derived Stem Cells. STEM CELLS.

[160] Horii M., Boyd T.K., Parast M.M., Crum C.P., Nucci M.R., Howitt B.E., Granter S.R., Parast M.M., Boyd T.K. (2018). Chapter 29—Placental Development and Complications of Previable Pregnancy. Diagnostic Gynecologic and Obstetric Pathology.

[161] Castellucci M., Kaufmann P. (2006). Basic structure of the villous trees. Pathology of the Human Placenta.

[162] Bhatnagar J., Rabeneck D.B., Martines R.B., Reagan-Steiner S., Ermias Y., Estetter L.B., Suzuki T., Ritter J., Keating M.K., Hale G. (2017). Zika virus RNA replication and persistence in brain and placental tissue. Emerg. Infect. Dis..

[163] Schwartz D.A. (2017). Viral infection, proliferation, and hyperplasia of Hofbauer cells and absence of inflammation characterize the placental pathology of fetuses with congenital Zika virus infection. Arch. Gynecol. Obstet..

[164] Aagaard K.M., Lahon A., Suter M.A., Arya R.P., Seferovic M.D., Vogt M.B., Hu M., Stossi F., Mancini M.A., Harris R.A. (2017). Primary Human Placental Trophoblasts are Permissive for Zika Virus (ZIKV) Replication. Sci. Rep..

[165] Liu S., DeLalio L.J., Isakson B.E., Wang T.T. (2016). AXL-Mediated Productive Infection of Human Endothelial Cells by Zika Virus. Circ. Res..

[166] Muthuraj P.G., Pattnaik A., Sahoo P.K., Islam M.T., Pattnaik A.K., Byrareddy S.N., Hanson C., Anderson Berry A., Kachman S.D., Natarajan S.K. (2021). Palmitoleate Protects against Zika Virus-Induced Placental Trophoblast Apoptosis. Biomedicines.

[167] Muthuraj P.G., Sahoo P.K., Kraus M., Bruett T., Annamalai A.S., Pattnaik A., Pattnaik A.K., Byrareddy S.N., Natarajan S.K. (2021). Zika virus infection induces endoplasmic reticulum stress and apoptosis in placental trophoblasts. Cell Death Discov..

[168] Tabata T., Petitt M., Puerta-Guardo H., Michlmayr D., Wang C., Fang-Hoover J., Harris E., Pereira L. (2016). Zika Virus Targets Different Primary Human Placental Cells, Suggesting Two Routes for Vertical Transmission. Cell Host Microbe.

[169] Weisblum Y., Oiknine-Djian E., Vorontsov O.M., Haimov-Kochman R., Zakay-Rones Z., Meir K., Shveiky D., Elgavish S., Nevo Y., Roseman M. (2017). Zika Virus Infects Early- and Midgestation Human Maternal Decidual Tissues, Inducing Distinct Innate Tissue Responses in the Maternal-Fetal Interface. J. Virol..

[170] Ades A.E., Soriano-Arandes A., Alarcon A., Bonfante F., Thorne C., Peckham C.S., Giaquinto C. (2021). Vertical transmission of Zika virus and its outcomes: A Bayesian synthesis of prospective studies. Lancet Infect. Dis..

[171] Robinson N., Mayorquin Galvan E.E., Zavala Trujillo I.G., Zavala-Cerna M.G. (2018). Congenital Zika syndrome: Pitfalls in the placental barrier. Rev. Med. Virol..

[172] Franca G.V., Schuler-Faccini L., Oliveira W.K., Henriques C.M., Carmo E.H., Pedi V.D., Nunes M.L., Castro M.C., Serruya S., Silveira M.F. (2016). Congenital Zika virus syndrome in Brazil: A case series of the first 1501 livebirths with complete investigation. Lancet.

[173] Soares de Souza A., Moraes Dias C., Braga F.D., Terzian A.C., Estofolete C.F., Oliani A.H., Oliveira G.H., Brandão de Mattos C.C., de Mattos L.C., Nogueira M.L. (2016). Fetal Infection by Zika Virus in the Third Trimester: Report of 2 Cases. Clin. Infect. Dis. Off. Publ. Infect. Dis. Soc. Am..

[174] Pomar L., Lambert V., Madec Y., Vouga M., Pomar C., Matheus S., Fontanet A., Panchaud A., Carles G., Baud D. (2020). Placental infection by Zika virus in French Guiana. Ultrasound Obstet. Gynecol..

[175] Adibi J.J., Marques E.T.A., Cartus A., Beigi R.H. (2016). Teratogenic effects of the Zika virus and the role of the placenta. Lancet.

[176] Chiu C.-F., Chu L.-W., Liao I.C., Simanjuntak Y., Lin Y.-L., Juan C.-C., Ping Y.-H. (2020). The Mechanism of the Zika Virus Crossing the Placental Barrier and the Blood-Brain Barrier. Front. Microbiol..

[177] Miranda J., Martin-Tapia D., Valdespino-Vazquez Y., Alarcon L., Espejel-Nunez A., Guzman-Huerta M., Munoz-Medina J.E., Shibayama M., Chavez-Munguia B., Estrada-Gutierrez G. (2019). Syncytiotrophoblast of Placentae from Women with Zika Virus Infection Has Altered Tight Junction Protein Expression and Increased Paracellular Permeability. Cells.

[178] Zhang Z.W., Li Z.L., Yuan S. (2016). The Role of Secretory Autophagy in Zika Virus Transfer through the Placental Barrier. Front. Cell. Infect. Microbiol..

[179] Leier H.C., Weinstein J.B., Kyle J.E., Lee J.-Y., Bramer L.M., Stratton K.G., Kempthorne D., Navratil A.R., Tafesse E.G., Hornemann T. (2020). A global lipid map defines a network essential for Zika virus replication. Nat. Commun..

[180] Molás R.B., Ribeiro M.R., Ramalho dos Santos M.J.C., Borbely A.U., Oliani D.V., Oliani A.H., Nadkarni S., Nogueira M.L., Moreli J.B., Oliani S.M. (2020). The involvement of annexin A1 in human placental response to maternal Zika virus infection. Antivir. Res..

[181] Priyamvada L., Quicke K.M., Hudson W.H., Onlamoon N., Sewatanon J., Edupuganti S., Pattanapanyasat K., Chokephaibulkit K., Mulligan M.J., Wilson P.C. (2016). Human antibody responses after dengue virus infection are highly cross-reactive to Zika virus. Proc. Natl. Acad. Sci. USA.

[182] Winkler C.W., Evans A.B., Carmody A.B., Peterson K.E. (2020). Placental myeloid cells protect against Zika virus vertical transmission in a murine model. J. Immunol..

[183] Amaral M.S., Goulart E., Caires-Júnior L.C., Morales-Vicente D.A., Soares-Schanoski A., Gomes R.P., de Oliveira Olberg G.G., Astray R.M., Kalil J.E., Zatz M. (2020). Differential gene expression elicited by ZIKV infection in trophoblasts from congenital Zika syndrome discordant twins. PLoS Negl. Trop. Dis..

[184] Suzukawa A.A., Zanluca C., Jorge N.A.N., de Noronha L., Koishi A.C., de Paula C.B.V., Rebutini P.Z., Nagashima S., Hansel-Frose A.F.F., Parreira V.S.C. (2020). Downregulation of IGF2 expression in third trimester placental tissues from Zika virus infected women in Brazil. J. Infect..

[185] Bordoni V., Lalle E., Colavita F., Baiocchini A., Nardacci R., Falasca L., Carletti F., Cimini E., Bordi L., Kobinger G. (2019). Rescue of Replication-Competent ZIKV Hidden in Placenta-Derived Mesenchymal Cells Long After the Resolution of the Infection. Open Forum Infect. Dis..

[186] Foo S.S., Chen W., Chan Y., Bowman J.W., Chang L.C., Choi Y., Yoo J.S., Ge J., Cheng G., Bonnin A. (2017). Asian Zika virus strains target CD14(+) blood monocytes and induce M2-skewed immunosuppression during pregnancy. Nat. Microbiol..

[187] Michlmayr D., Andrade P., Gonzalez K., Balmaseda A., Harris E. (2017). CD14(+)CD16(+) monocytes are the main target of Zika virus infection in peripheral blood mononuclear cells in a paediatric study in Nicaragua. Nat. Microbiol..

[188] Hirsch A.J., Roberts V.H.J., Grigsby P.L., Haese N., Schabel M.C., Wang X., Lo J.O., Liu Z., Kroenke C.D., Smith J.L. (2018). Zika virus infection in pregnant rhesus macaques causes placental dysfunction and immunopathology. Nat. Commun..

[189] Miner J.J., Cao B., Govero J., Smith A.M., Fernandez E., Cabrera O.H., Garber C., Noll M., Klein R.S., Noguchi K.K. (2016). Zika Virus Infection during Pregnancy in Mice Causes Placental Damage and Fetal Demise. Cell.

[190] Yockey L.J., Jurado K.A., Arora N., Millet A., Rakib T., Milano K.M., Hastings A.K., Fikrig E., Kong Y., Horvath T.L. (2018). Type I interferons instigate fetal demise after Zika virus infection. Sci. Immunol..

[191] Gorman M.J., Caine E.A., Zaitsev K., Begley M.C., Weger-Lucarelli J., Uccellini M.B., Tripathi S., Morrison J., Yount B.L., Dinnon K.H. (2018). An Immunocompetent Mouse Model of Zika Virus Infection. Cell Host Microbe.

[192] Rufo N., Garg A.D., Agostinis P. (2017). The Unfolded Protein Response in Immunogenic Cell Death and Cancer Immunotherapy. Trends Cancer.

[193] Corazzari M., Gagliardi M., Fimia G.M., Piacentini M. (2017). Endoplasmic Reticulum Stress, Unfolded Protein Response, and Cancer Cell Fate. Front. Oncol..

[194] Blazquez A.B., Escribano-Romero E., Merino-Ramos T., Saiz J.C., Martin-Acebes M.A. (2014). Stress responses in flavivirus-infected cells: Activation of unfolded protein response and autophagy. Front. Microbiol..

[195] Rossignol E.D., Peters K.N., Connor J.H., Bullitt E. (2017). Zika virus induced cellular remodelling. Cell. Microbiol..

[196] Gladwyn-Ng I., Cordon-Barris L., Alfano C., Creppe C., Couderc T., Morelli G., Thelen N., America M., Bessieres B., Encha-Razavi F. (2018). Stress-induced unfolded protein response contributes to Zika virus-associated microcephaly. Nat. Neurosci..

[197] Alfano C., Gladwyn-Ng I., Couderc T., Lecuit M., Nguyen L. (2019). The Unfolded Protein Response: A Key Player in Zika Virus-Associated Congenital Microcephaly. Front. Cell Neurosci..

[198] Turpin J., Frumence E., Harrabi W., Haddad J.G., El Kalamouni C., Desprès P., Krejbich-Trotot P., Viranaïcken W. (2020). Zika virus subversion of chaperone GRP78/BiP expression in A549 cells during UPR activation. Biochimie.

[199] Tan Z., Zhang W., Sun J., Fu Z., Ke X., Zheng C., Zhang Y., Li P., Liu Y., Hu Q. (2018). ZIKV infection activates the IRE1-XBP1 and ATF6 pathways of unfolded protein response in neural cells. J. Neuroinflammation.

[200] Hu R., Clarke R., Clarke R. (2019). Roles of Spliced and Unspliced XBP1 in Breast Cancer. The Unfolded Protein Response in Cancer.

[201] Huang Y., Lin Q., Huo Z., Chen C., Zhou S., Ma X., Gao H., Lin Y., Li X., He J. (2020). Inositol-Requiring Enzyme 1α Promotes Zika Virus Infection through Regulation of Stearoyl Coenzyme A Desaturase 1-Mediated Lipid Metabolism. J. Virol..

[202] Zhang L., Wang A. (2012). Virus-induced ER stress and the unfolded protein response. Front. Plant Sci..

[203] Hu H., Tian M., Ding C., Yu S. (2019). The C/EBP Homologous Protein (CHOP) Transcription Factor Functions in Endoplasmic Reticulum Stress-Induced Apoptosis and Microbial Infection. Front. Immunol..

[204] Ohoka N., Yoshii S., Hattori T., Onozaki K., Hayashi H. (2005). TRB3, a novel ER stress-inducible gene, is induced via ATF4-CHOP pathway and is involved in cell death. Embo J..

[205] Rao J., Zhang C., Wang P., Lu L., Qian X., Qin J., Pan X., Li G., Wang X., Zhang F. (2015). C/EBP homologous protein (CHOP) contributes to hepatocyte death via the promotion of ERO1α signalling in acute liver failure. Biochem. J..

[206] Adler H.T., Chinery R., Wu D.Y., Kussick S.J., Payne J.M., Fornace A.J., Tkachuk D.C. (1999). Leukemic HRX fusion proteins inhibit GADD34-induced apoptosis and associate with the GADD34 and hSNF5/INI1 proteins. Mol. Cell. Biol..

[207] Szegezdi E., Logue S.E., Gorman A.M., Samali A. (2006). Mediators of endoplasmic reticulum stress-induced apoptosis. EMBO Rep..

[208] Ceballos-Olvera I., Chavez-Salinas S., Medina F., Ludert J.E., del Angel R.M. (2010). JNK phosphorylation, induced during dengue virus infection, is important for viral infection and requires the presence of cholesterol. Virology.

[209] Lennemann N.J., Coyne C.B. (2017). Dengue and Zika viruses subvert reticulophagy by NS2B3-mediated cleavage of FAM134B. Autophagy.

[210] Cao H., Gerhold K., Mayers J.R., Wiest M.M., Watkins S.M., Hotamisligil G.S. (2008). Identification of a lipokine, a lipid hormone linking adipose tissue to systemic metabolism. Cell.

[211] Hodson L., Karpe F. (2013). Is there something special about palmitoleate?. Curr. Opin. Clin. Nutr. Metab. Care.

[212] de Souza C.O., Vannice G.K., Rosa Neto J.C., Calder P.C. (2018). Is Palmitoleic Acid a Plausible Nonpharmacological Strategy to Prevent or Control Chronic Metabolic and Inflammatory Disorders?. Mol. Nutr. Food Res..

[213] Frigolet M.E., Gutiérrez-Aguilar R. (2017). The Role of the Novel Lipokine Palmitoleic Acid in Health and Disease. Adv. Nutr..

[214] Mozaffarian D., de Oliveira Otto M.C., Lemaitre R.N., Fretts A.M., Hotamisligil G., Tsai M.Y., Siscovick D.S., Nettleton J.A. (2013). trans-Palmitoleic acid, other dairy fat biomarkers, and incident diabetes: The Multi-Ethnic Study of Atherosclerosis (MESA). Am. J. Clin. Nutr..

[215] Cunningham E. (2015). What Are n-7 Fatty Acids and Are There Health Benefits Associated with Them?. J. Acad. Nutr. Diet..

[216] Hu W., Fitzgerald M., Topp B., Alam M., O’Hare T.J. (2019). A review of biological functions, health benefits, and possible de novo biosynthetic pathway of palmitoleic acid in macadamia nuts. J. Funct. Foods.

[217] Carrillo W., Carpio C., Morales D., Vilcacundo E., Alvarez M. (2017). Fatty acids composition in macadamia seed oil (Macadamia integrifolia) from Ecuador. Asian J. Pharm. Clin. Res..

[218] O’Hare T.J., Trieu H.H., Topp B., Russell D., Pun S., Torrisi C., Liu D. (2019). Assessing fatty acid profiles of macadamia nuts. HortScience.

[219] Rengel A., Pérez E., Piombo G., Ricci J., Servent A., Tapia M.S., Gibert O., Montet D. (2015). Lipid profile and antioxidant activity of macadamia nuts (Macadamia integrifolia) cultivated in Venezuela. Nat. Sci..

[220] Foryst-Ludwig A., Kreissl M.C., Benz V., Brix S., Smeir E., Ban Z., Januszewicz E., Salatzki J., Grune J., Schwanstecher A.K. (2015). Adipose Tissue Lipolysis Promotes Exercise-induced Cardiac Hypertrophy Involving the Lipokine C16:1n7-Palmitoleate. J. Biol. Chem..

[221] Burns T.A., Duckett S.K., Pratt S.L., Jenkins T.C. (2012). Supplemental palmitoleic (C16:1 cis-9) acid reduces lipogenesis and desaturation in bovine adipocyte cultures. J. Anim. Sci..

[222] Duckett S.K., Volpi-Lagreca G., Alende M., Long N.M. (2014). Palmitoleic acid reduces intramuscular lipid and restores insulin sensitivity in obese sheep. Diabetes Metab. Syndr. Obes..

[223] Griel A.E., Cao Y., Bagshaw D.D., Cifelli A.M., Holub B., Kris-Etherton P.M. (2008). A macadamia nut-rich diet reduces total and LDL-cholesterol in mildly hypercholesterolemic men and women. J. Nutr..

[224] Garg M.L., Blake R.J., Wills R.B. (2003). Macadamia nut consumption lowers plasma total and LDL cholesterol levels in hypercholesterolemic men. J. Nutr..

[225] Yang Z.H., Pryor M., Noguchi A., Sampson M., Johnson B., Pryor M., Donkor K., Amar M., Remaley A.T. (2019). Dietary Palmitoleic Acid Attenuates Atherosclerosis Progression and Hyperlipidemia in Low-Density Lipoprotein Receptor-Deficient Mice. Mol. Nutr. Food Res..

[226] Kenny L.C., Baker P.N., Kendall D.A., Randall M.D., Dunn W.R. (2002). The role of gap junctions in mediating endothelium-dependent responses to bradykinin in myometrial small arteries isolated from pregnant women. Br. J. Pharmacol..

[227] Tricò D., Mengozzi A., Nesti L., Hatunic M., Gabriel Sanchez R., Konrad T., Lalić K., Lalić N.M., Mari A., Natali A. (2020). Circulating palmitoleic acid is an independent determinant of insulin sensitivity, beta cell function and glucose tolerance in non-diabetic individuals: A longitudinal analysis. Diabetologia.

[228] Chan K.L., Pillon N.J., Sivaloganathan D.M., Costford S.R., Liu Z., Théret M., Chazaud B., Klip A. (2015). Palmitoleate reverses high fat-induced proinflammatory macrophage polarization via AMP-activated protein kinase (AMPK). J. Biol. Chem..

[229] Morse N. (2015). Are some health benefits of palmitoleic acid supplementation due to its effects on 5′ adenosine monophosphate-activated protein kinase (AMPK)?. Lipid Technol..

[230] Souza C.O., Teixeira A.A.S., Biondo L.A., Silveira L.S., de Souza Breda C.N., Braga T.T., Camara N.O.S., Belchior T., Festuccia W.T., Diniz T.A. (2020). Palmitoleic acid reduces high fat diet-induced liver inflammation by promoting PPAR-γ-independent M2a polarization of myeloid cells. Biochim. Biophys. Acta (BBA)-Mol. Cell Biol. Lipids.

[231] Souza C.O., Teixeira A.A.S., Lima E.A., Batatinha H.A.P., Gomes L.M., Carvalho-Silva M., Mota I.T., Streck E.L., Hirabara S.M., Neto J.C.R. (2014). Palmitoleic Acid (N-7) Attenuates the Immunometabolic Disturbances Caused by a High-Fat Diet Independently of PPAR*α*. Mediat. Inflamm..

[232] Bolsoni-Lopes A., Festuccia W.T., Farias T.S., Chimin P., Torres-Leal F.L., Derogis P.B., de Andrade P.B., Miyamoto S., Lima F.B., Curi R. (2013). Palmitoleic acid (n-7) increases white adipocyte lipolysis and lipase content in a PPARα-dependent manner. Am. J. Physiol. Endocrinol. Metab..

[233] Ferchaud-Roucher V., Barner K., Jansson T., Powell T.L. (2019). Maternal obesity results in decreased syncytiotrophoblast synthesis of palmitoleic acid, a fatty acid with anti-inflammatory and insulin-sensitizing properties. FASEB J..

[234] Weimann E., Silva M.B.B., Murata G.M., Bortolon J.R., Dermargos A., Curi R., Hatanaka E. (2018). Topical anti-inflammatory activity of palmitoleic acid improves wound healing. PLoS ONE.

[235] Yang Z.-H., Takeo J., Katayama M. (2013). Oral administration of omega-7 palmitoleic acid induces satiety and the release of appetite-related hormones in male rats. Appetite.

[236] Bjermo H., Risérus U. (2010). Role of hepatic desaturases in obesity-related metabolic disorders. Curr. Opin. Clin. Nutr. Metab. Care.

[237] Djoussé L., Matthan N.R., Lichtenstein A.H., Gaziano J.M. (2012). Red Blood Cell Membrane Concentration of cis-Palmitoleic and cis-Vaccenic Acids and Risk of Coronary Heart Disease. Am. J. Cardiol..

[238] Sands J.A. (1977). Inactivation and inhibition of replication of the enveloped bacteriophage phi6 by fatty acids. Antimicrob. Agents Chemother..

